# Supplementary material for: Assessing the Impact of Antimicrobial Resistance Awareness Interventions Among Schoolchildren in Bangladesh
Source: Antibiotics (Basel). 2025 Sep 29;14(10):979. doi: 10.3390/antibiotics14100979 (PMC12561141; doi:10.3390/antibiotics14100979)
Supplement: Supplementary file 1 [file antibiotics-14-00979-s001.zip › File S1.pdf]

Assessing the impact of antimicrobial resistance awareness interventions among school children in Bangladesh

Respondent's Name:

School:

Class:

Division (if):

Section:

Shift:

Roll:

Present address:

Gender: Male / Female / Other

Religion:

Age:

Occupation of Father:

Occupation of Mother:

How many brothers and sisters do you have?:

1. **Have you ever heard of antimicrobial resistance (AMR)?**
   - Yes
   - No
2. **From where have you heard about antimicrobial resistance (AMR)?**

3. **How can you identify whether a medicine is an antibiotic?**

4. **If you have antibiotics at home that you previously took when you were sick, would you take them again the next time you become ill?**
   - Yes
   - No
   - Don't know

5. **It is acceptable to share antibiotics with family members or friends if they have similar symptoms.**
   - Yes
   - No
   - Don’t know
6. **If I have a mild infection (e.g., fever, diarrhea, etc.), I should use antibiotics instead of relying on natural remedies or rest.**
   - Yes
   - No
   - Don’t know
7. **Taking higher doses of antibiotics or taking them for a longer period will help me recover faster.**
   - Yes
   - No
   - Don’t know
8. **If antibiotics become ineffective over time for some individuals, will they also become ineffective for people around them?**
   - Yes
   - No
   - Don’t know
9. **If you feel better after taking half of the prescribed course of antibiotics, will you continue taking them until the course is completed as per the doctor’s prescription?**
   - Yes
   - No
   - Don’t know

10. **Do you believe that any antibiotic is effective in curing all types of infections, regardless of the specific type or cause of infection?**

• Yes

• No

• Don't know

11. **Do you think it is beneficial to take antibiotics directly from a pharmacy without consulting a doctor?**

• Yes

• No

• Don't know
