# Supplementary material for: Assessing the Impact of Antimicrobial Resistance Awareness Interventions Among Schoolchildren in Bangladesh
Source: Antibiotics (Basel). 2025 Sep 29;14(10):979. doi: 10.3390/antibiotics14100979 (PMC12561141; doi:10.3390/antibiotics14100979)
Supplement: Supplementary file 1 [file antibiotics-14-00979-s001.zip › File S2.pdf]

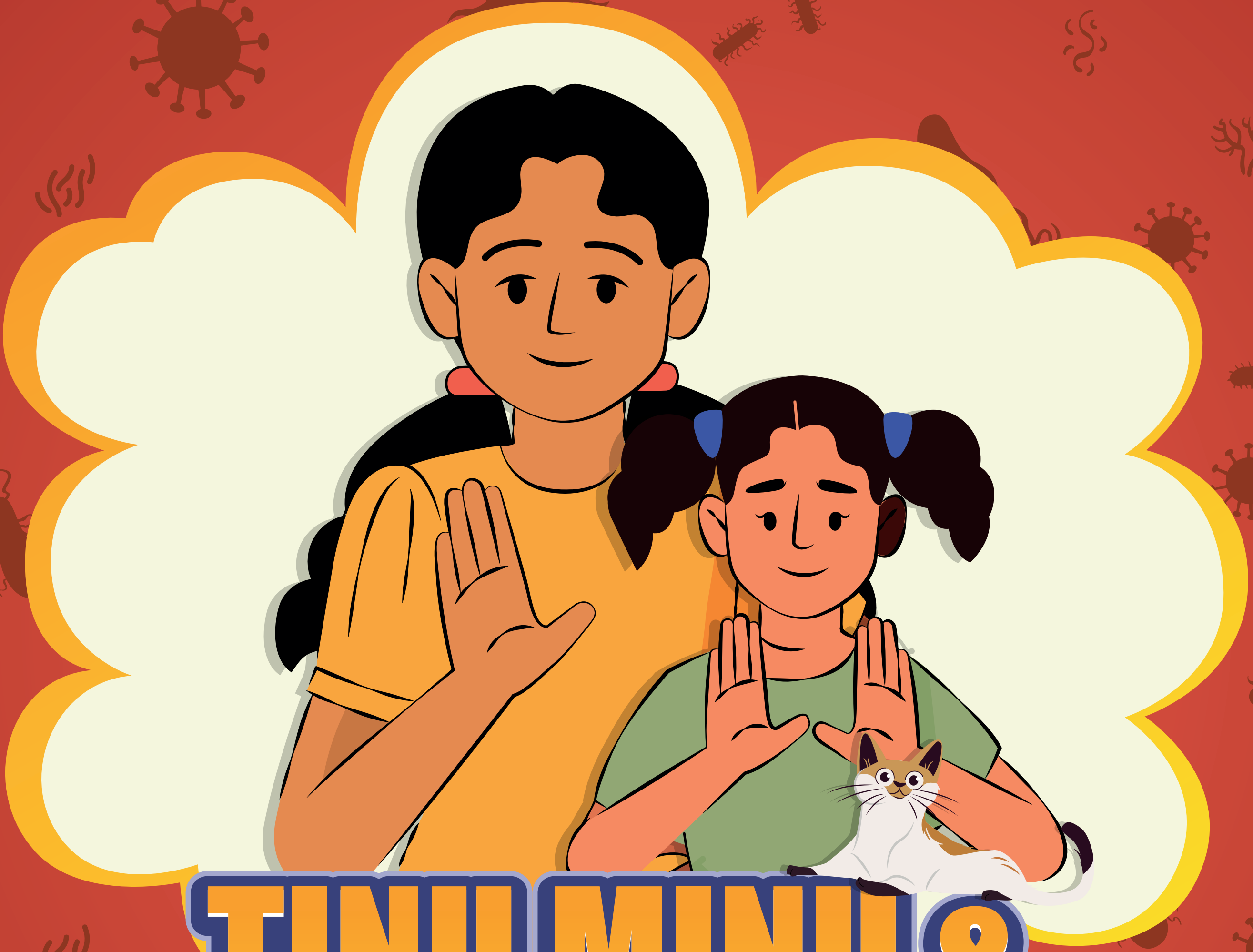

TINU MINU & SUPER BUG

An Awareness Story

First release
November, 2022

Editorial Board
Major General Mohammad Yousuf, Director General, Directorate General of Drug Administration.
Mr. Md. Mostafizur Rahman, Director (cc), Directorate General of Drug Administration.
Ms. S. M. Sabrina Yesmin, Assistant Director, Directorate General of Drug Administration.
Mr. ATM Golam Kibria Khan, Assistant Director, Directorate General of Drug Administration.
Mr. Md. Kamrul Hasan, Assistant Director, Directorate General of Drug Administration.

Narrator
(1) Tinu-Minu and Super Bug: Ms. S. M. Sabrina Yesmin, Assistant Director, DGDA.
(2) Thoughts of Tapa-Gopi: Ms. Umme Habiba, National Consultant-AMR, WHO-Bangladesh.

Translator
Mr. Paritosh Chakma
National Consultant- Essential Drugs and other Medicines
WHO Country Office Bangladesh

Cover and Graphics
Ms. S. M. Shanzida Yeasmin, Dhaka, Bangladesh.

The Government of the People's Republic of Bangladesh
Directorate General of Drug Administration, Health Service Division
Ministry of Health and Family Welfare

Introduction

An even bigger epidemic than Covid-19 awaits us is antimicrobial resistance (AMR). The World Health Organization has declared antimicrobial resistance (AMR) as one of human civilisation's top 10 health threats. Currently 12 lakh 70 thousand people die annually due to antimicrobial resistance. If this continues, one crore people will die every year in 2050.

Naturally occurring antimicrobial drugs can kill various microorganisms (eg, bacteria, viruses, fungi, parasites). But the special condition in which the antimicrobial medicine cannot destroy all these microorganisms or fails, that condition is called antimicrobial resistance. Antimicrobial drugs are antibiotics, antivirals, antifungals and antiparasitics.

The main causes of antimicrobial resistance are unnecessary or non-prescription antimicrobial drugs, especially voluntary consumption of antibiotics, not completing the full course of antibiotics, use of antibiotics in animal and fish feed (feed) or treatment.

We must be aware of protecting our future generation from this coming pandemic. This “comics book” has been prepared by the Directorate General of Drug Administration so that children, adolescents, and youth can also understand this clearly, and they can make their parents, relatives and neighbors aware.

Table of Contents

1. Tinu Minu and Super Bug
2. Thoughts of Tapa-Gopi

Introduction

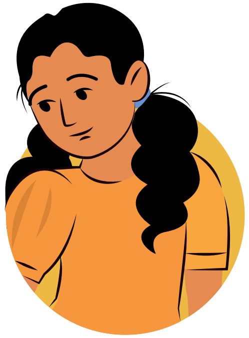
Tinu

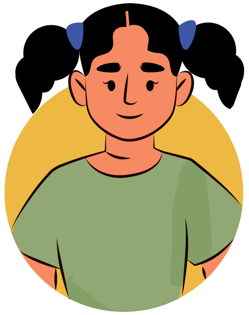
Minu

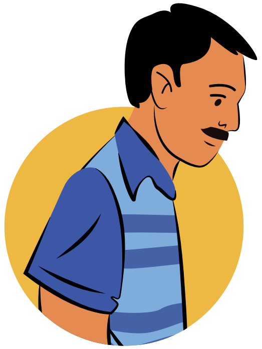
Father

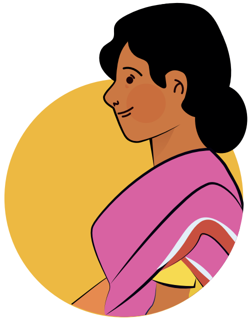
Mother

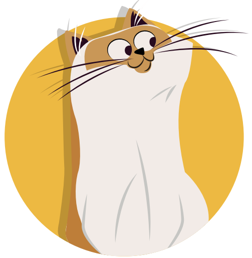
Tultul

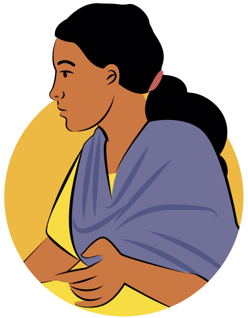
Nilu apa

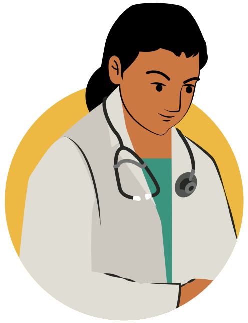
Doctor apa

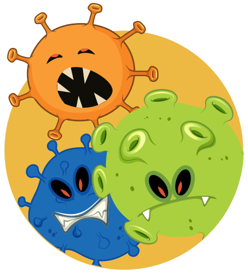
The virus

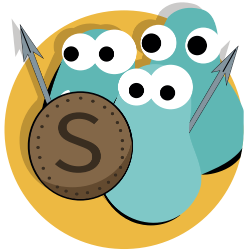

Immune system

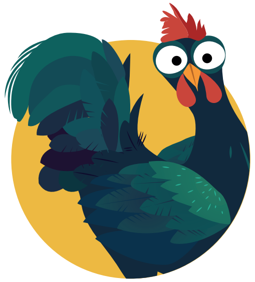
Tapa chicken

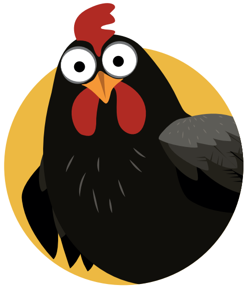
Gopi chicken

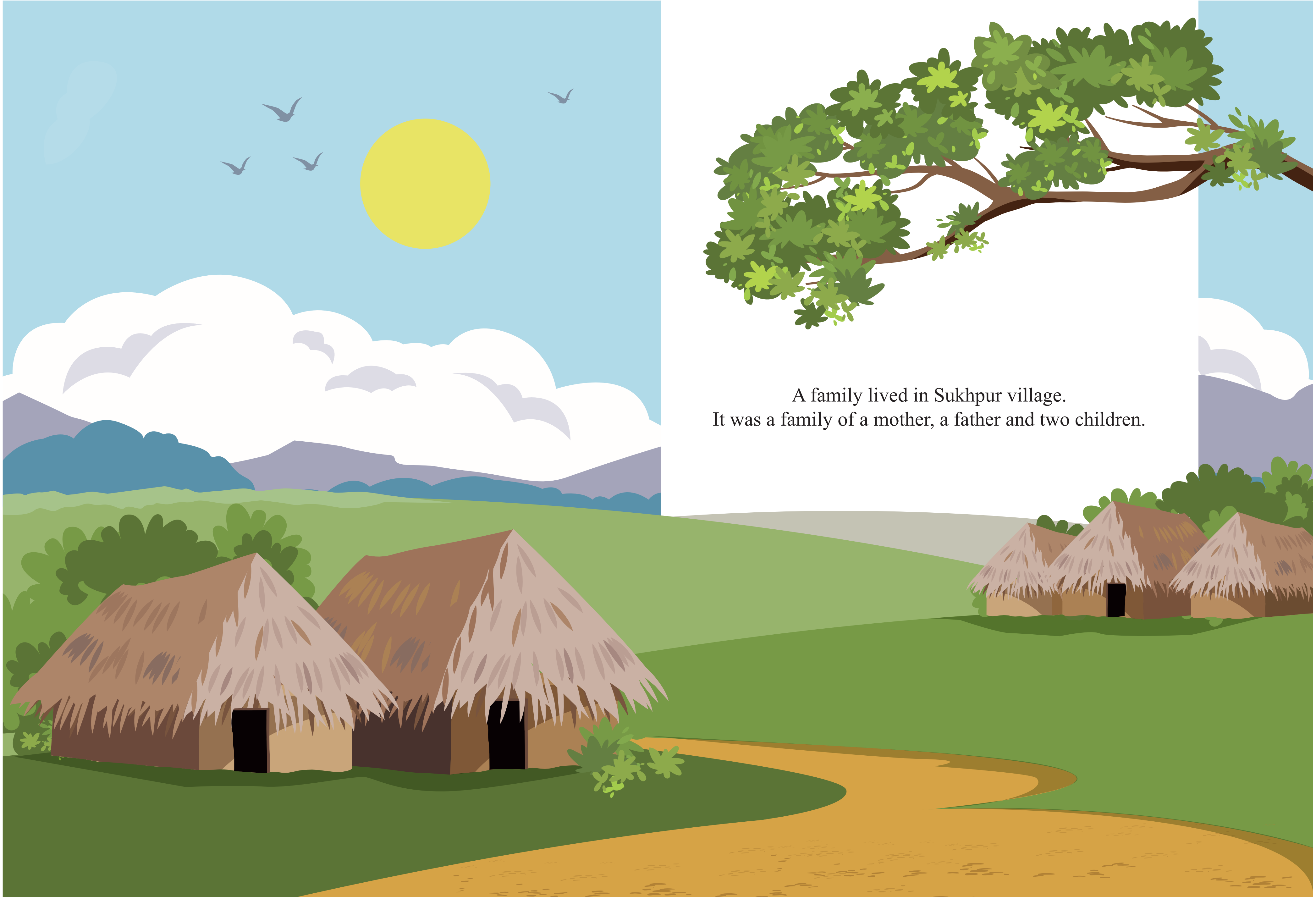

A family lived in Sukhpur village.
It was a family of a mother, a father and two children.

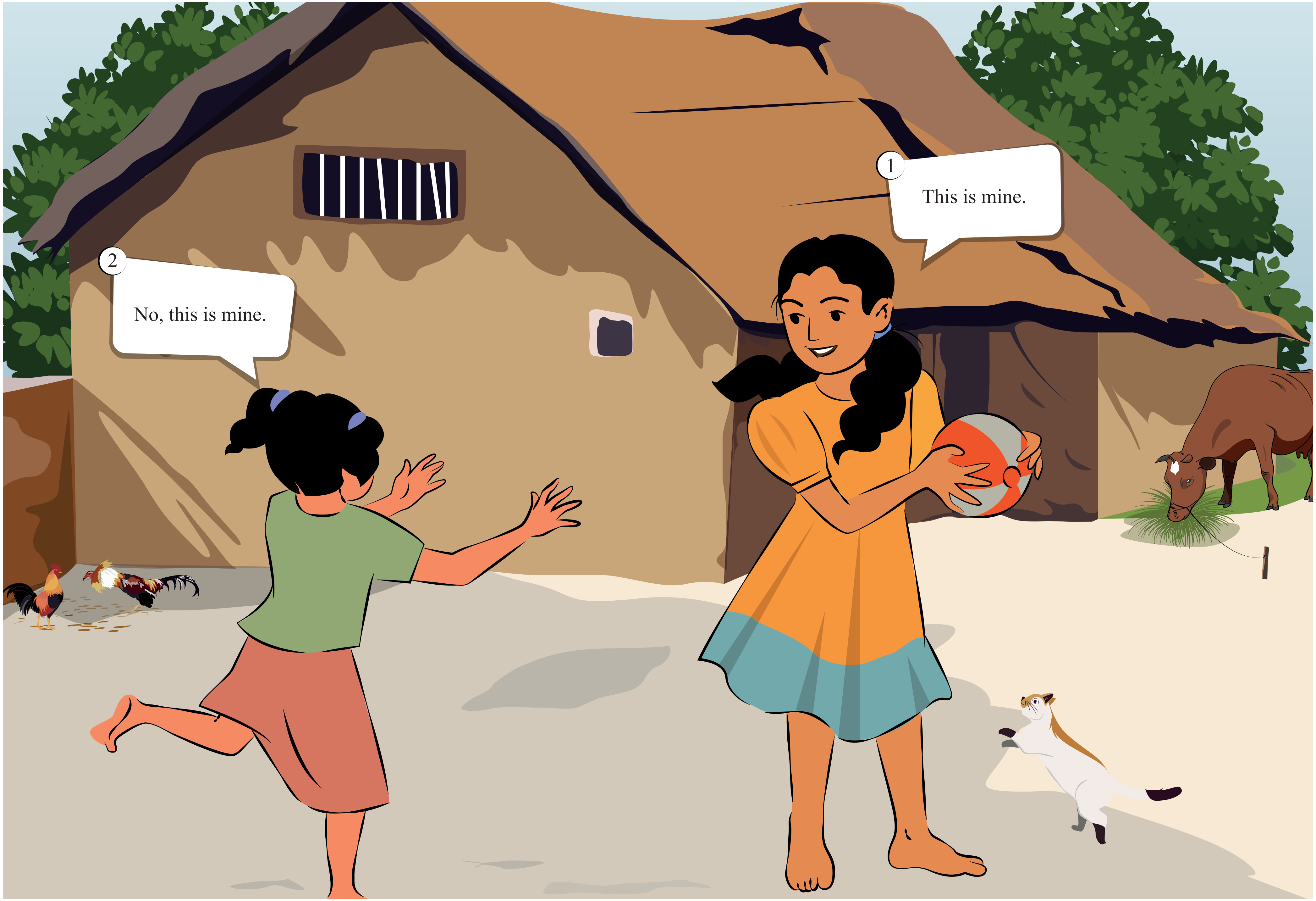
1
This is mine.
2
No, this is mine.

Mother has been ill for several days.

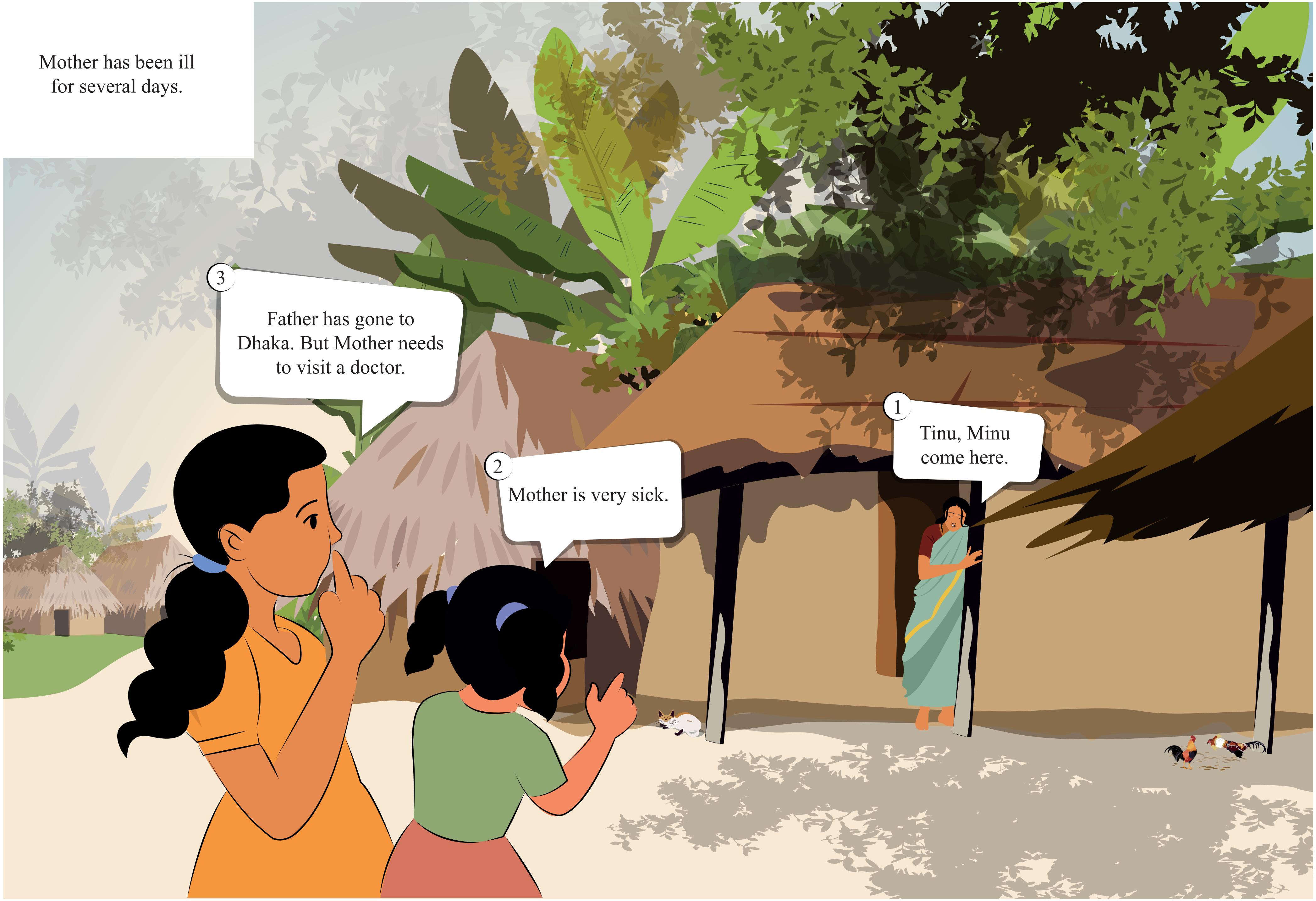

The Mother is being infected by viruses.

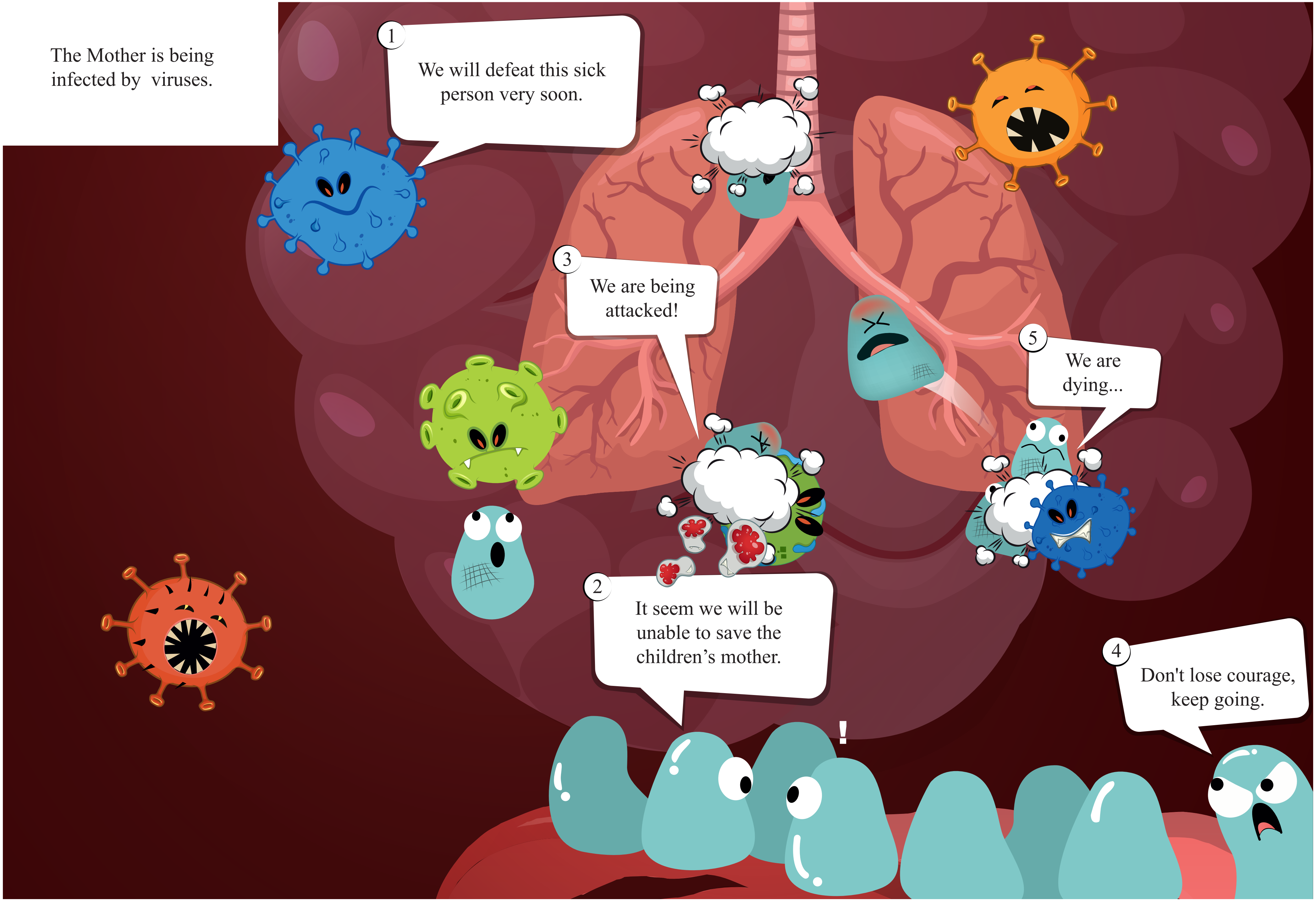

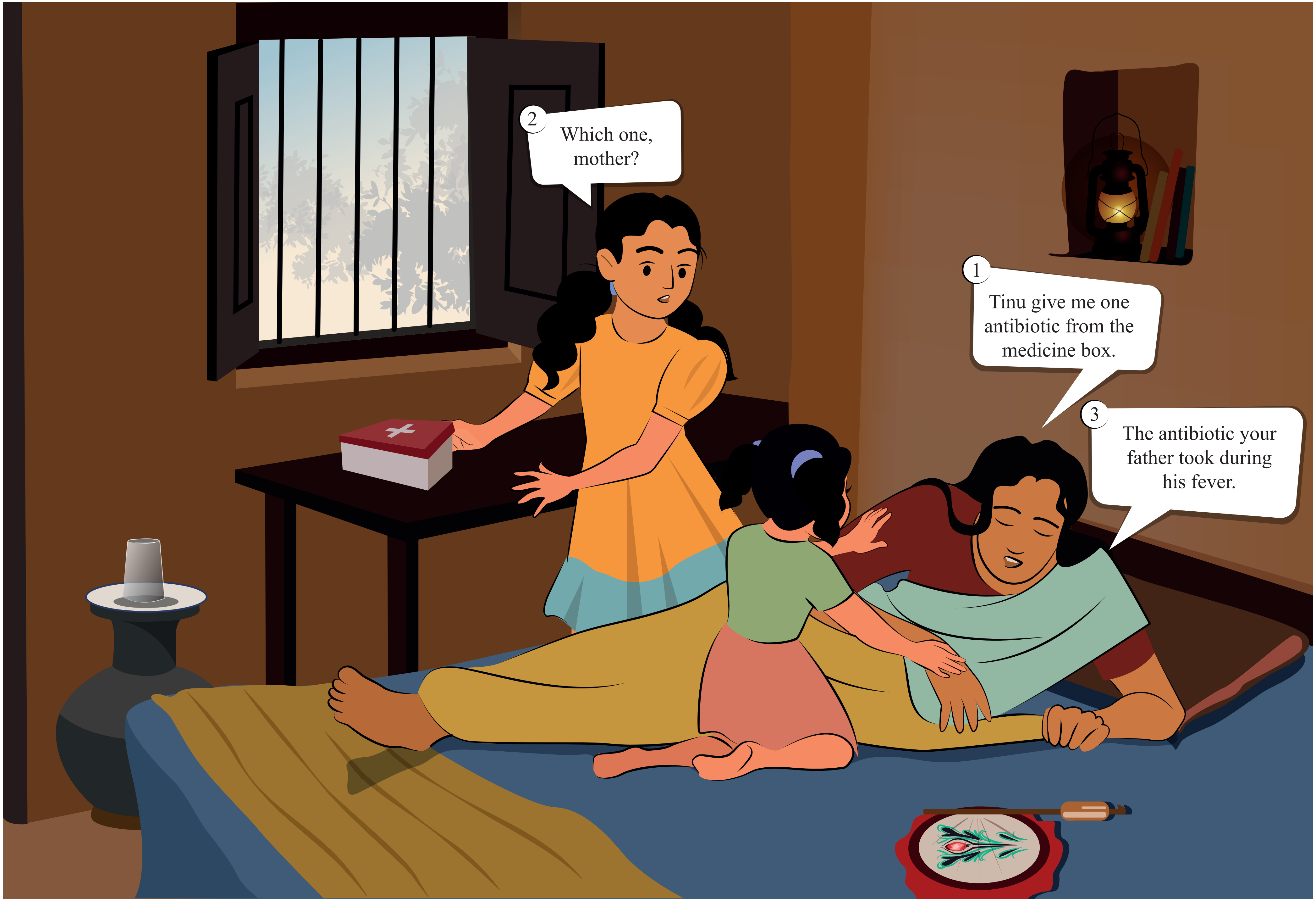
1
Tinu give me one antibiotic from the medicine box.
2
Which one, mother?
3
The antibiotic your father took during his fever.

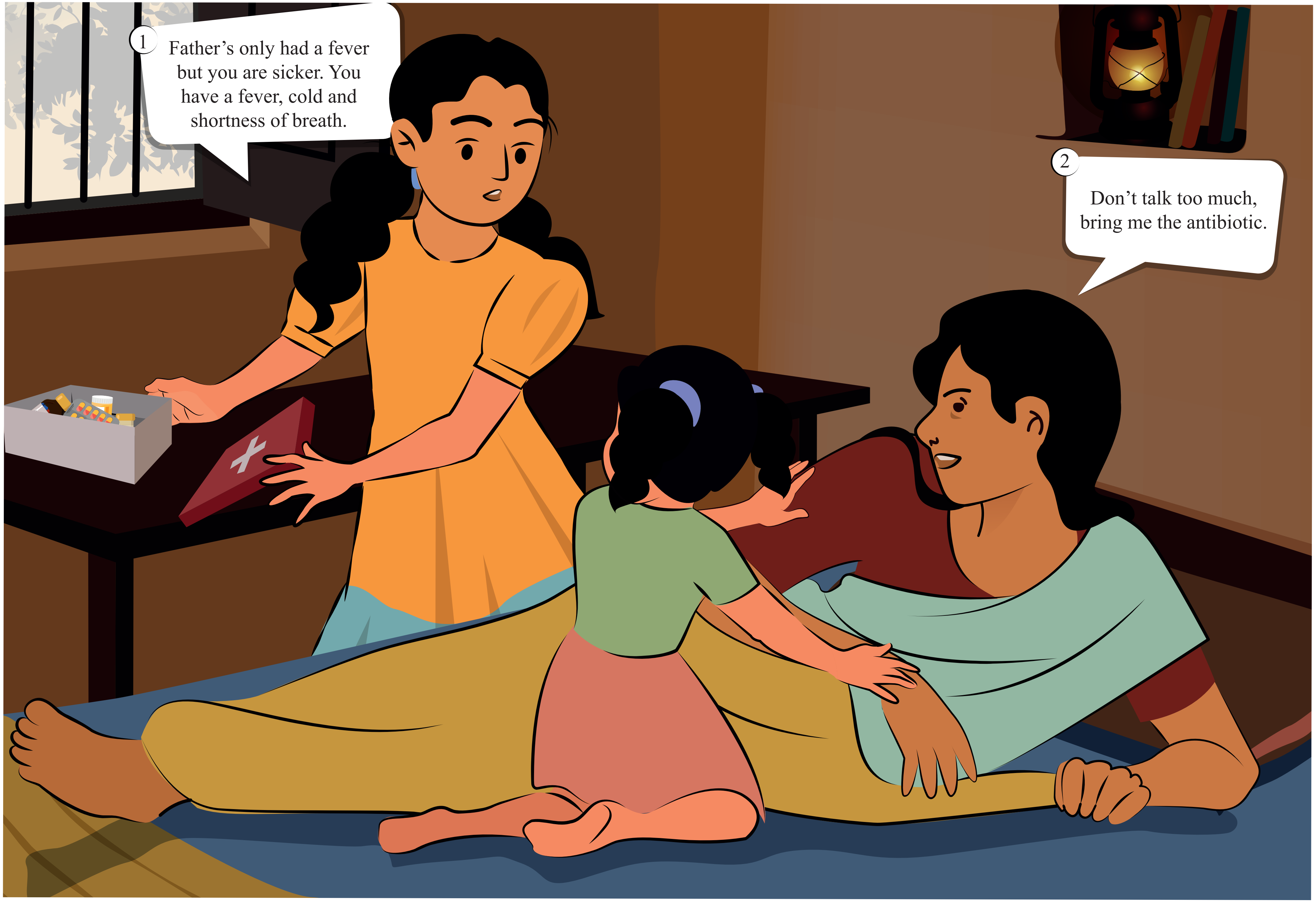
1
Father's only had a fever but you are sicker. You have a fever, cold and shortness of breath.
2
Don't talk too much, bring me the antibiotic.

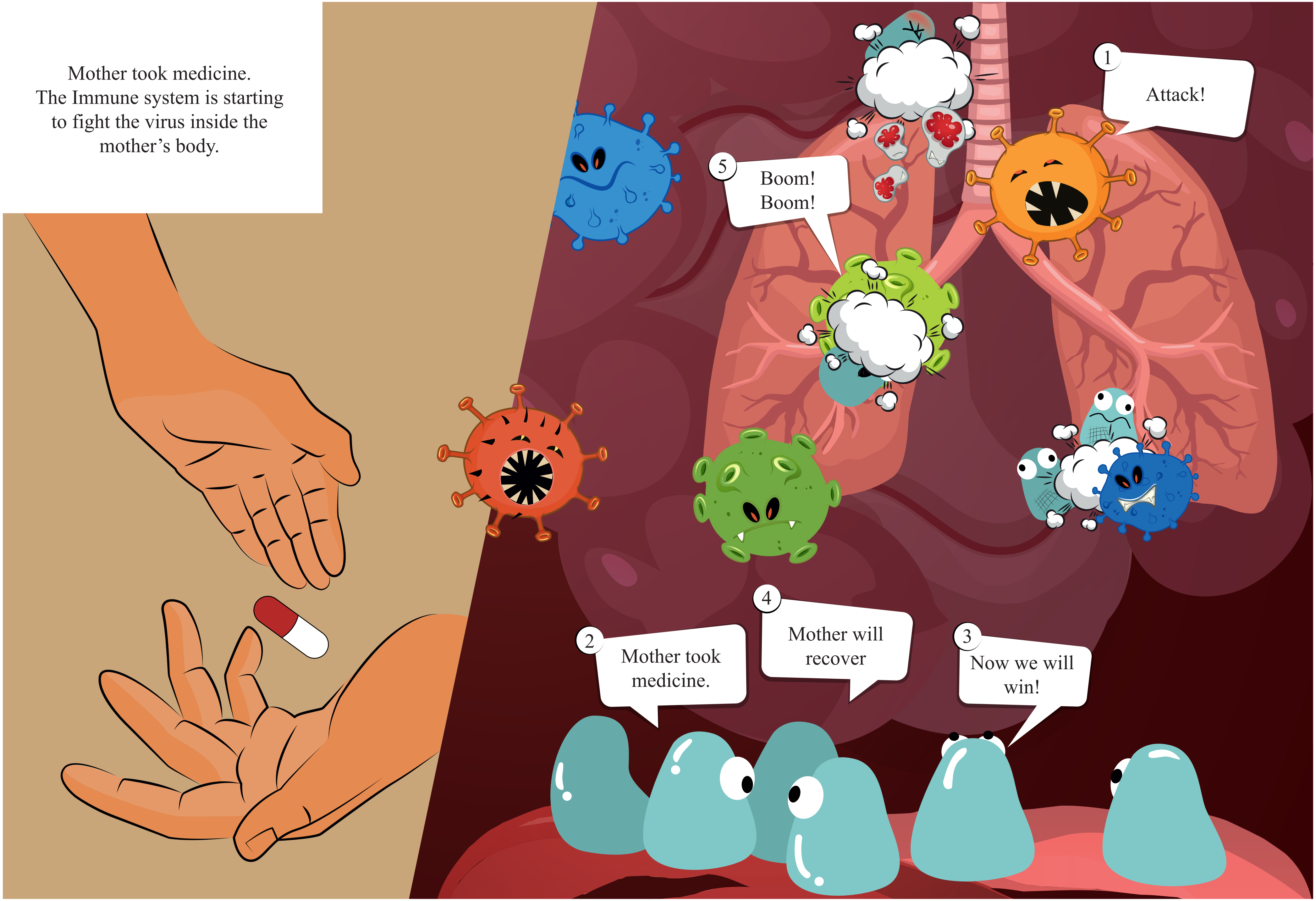
Mother took medicine.
The Immune system is starting
to fight the virus inside the
mother's body.
1
Attack!
2
Mother took
medicine.
3
Now we will
win!
4
Mother will
recover
5
Boom!
Boom!

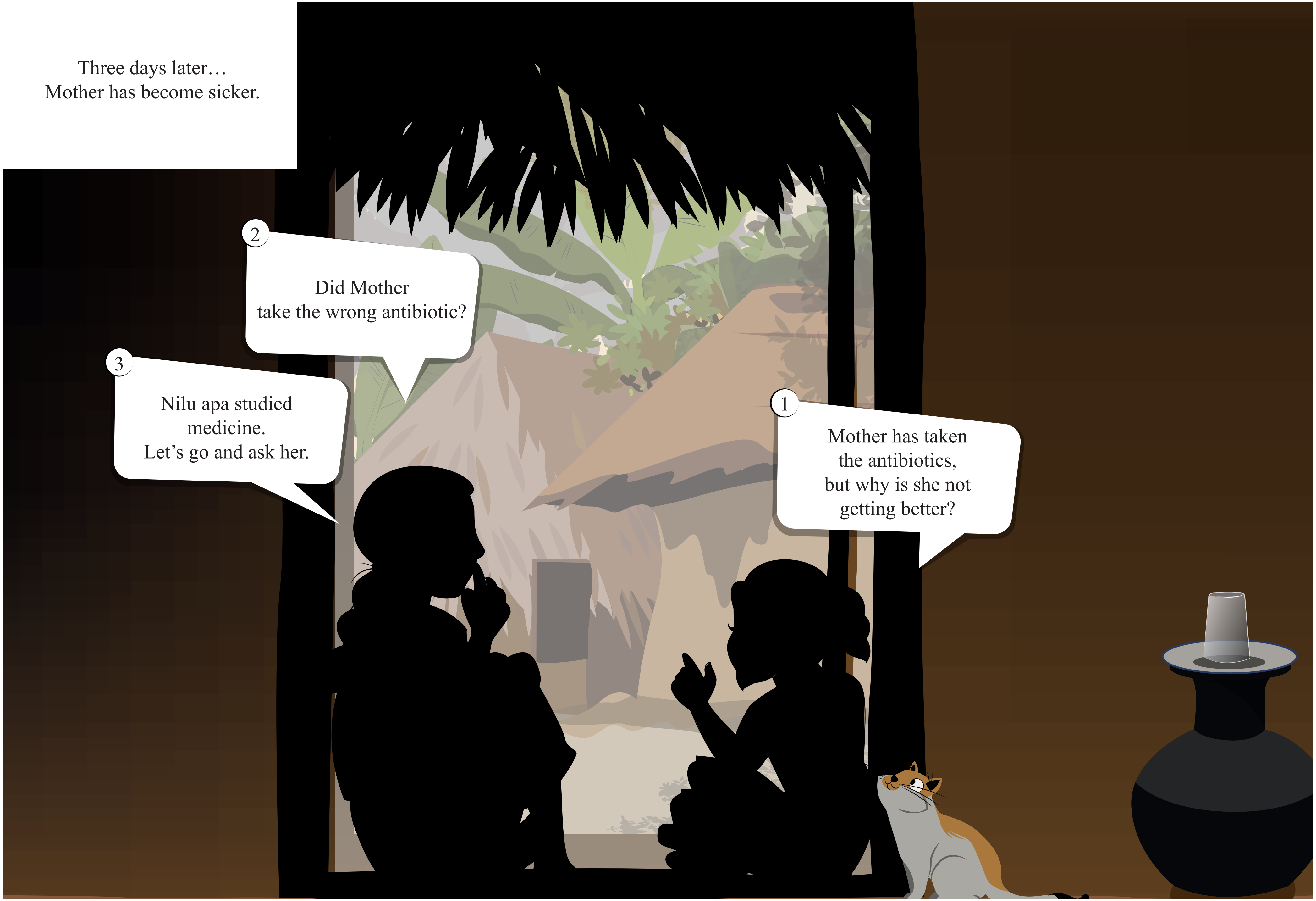
Three days later…
Mother has become sicker.
Mother has taken the antibiotics, but why is she not getting better?
Did Mother take the wrong antibiotic?
Nilu apa studied medicine. Let's go and ask her.

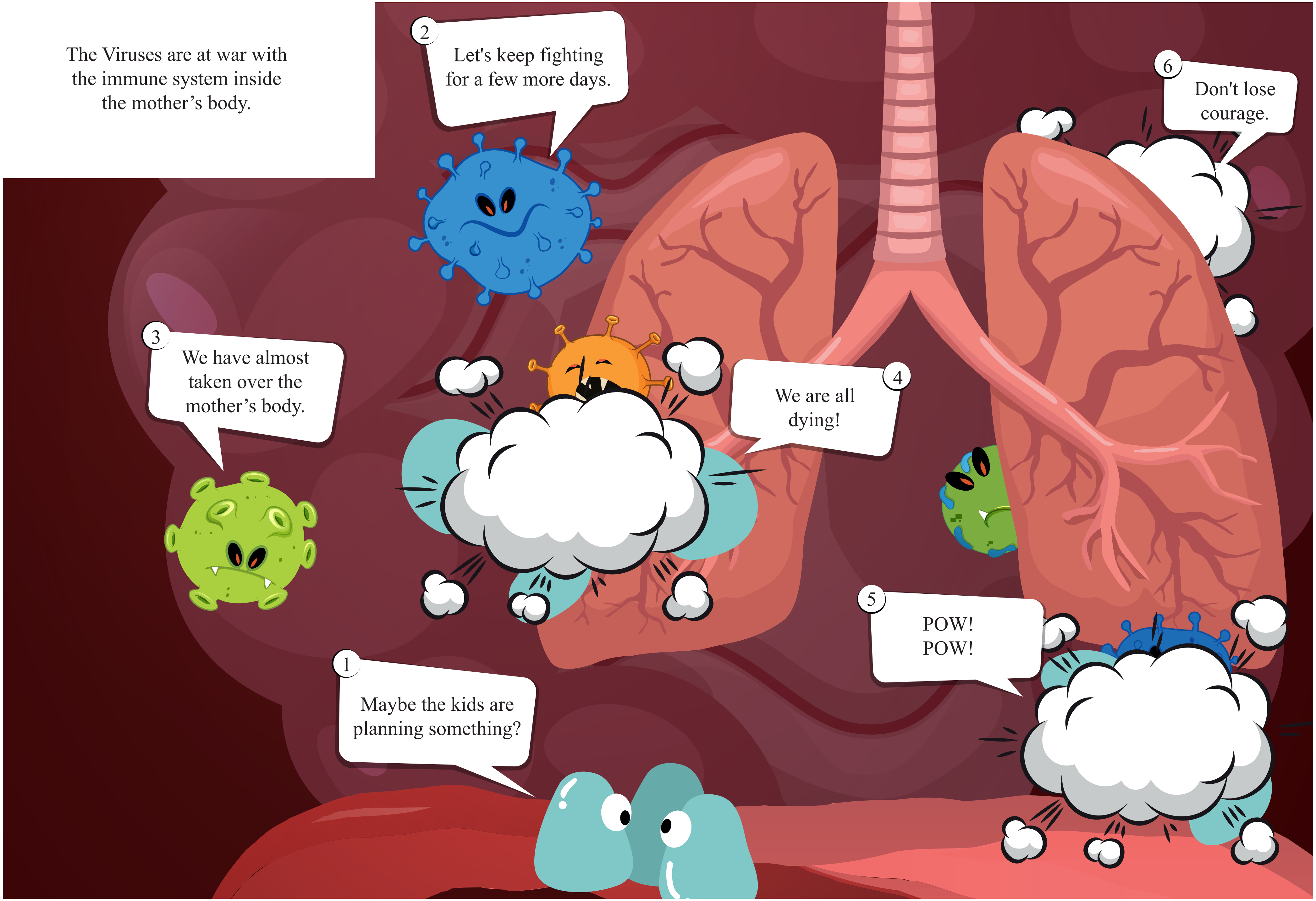
The Viruses are at war with the immune system inside the mother’s body.
1 Maybe the kids are planning something?
2 Let's keep fighting for a few more days.
3 We have almost taken over the mother’s body.
4 We are all dying!
5 POW! POW!
6 Don't lose courage.

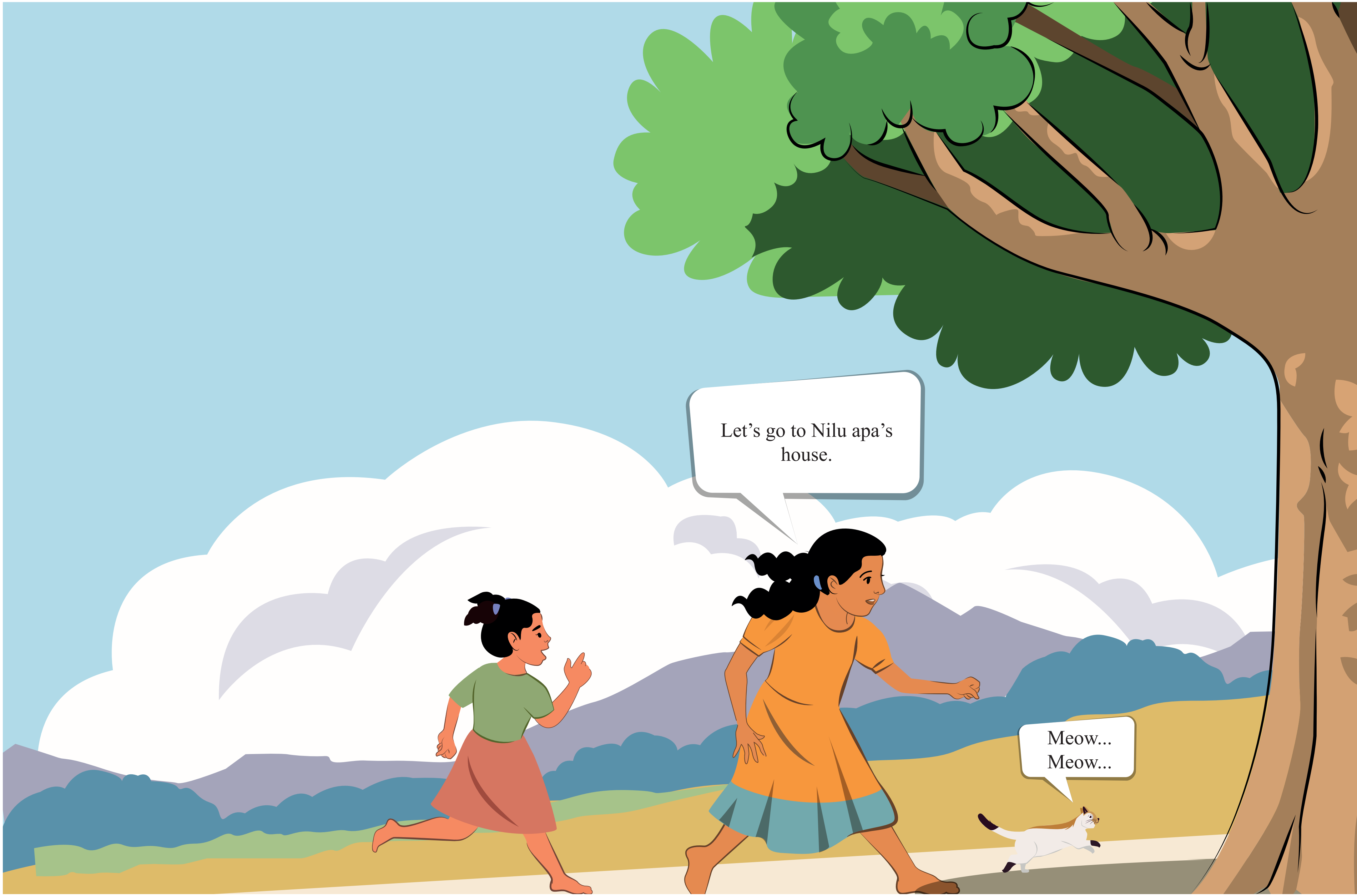
Let’s go to Nilu apa’s house.
Meow... Meow...

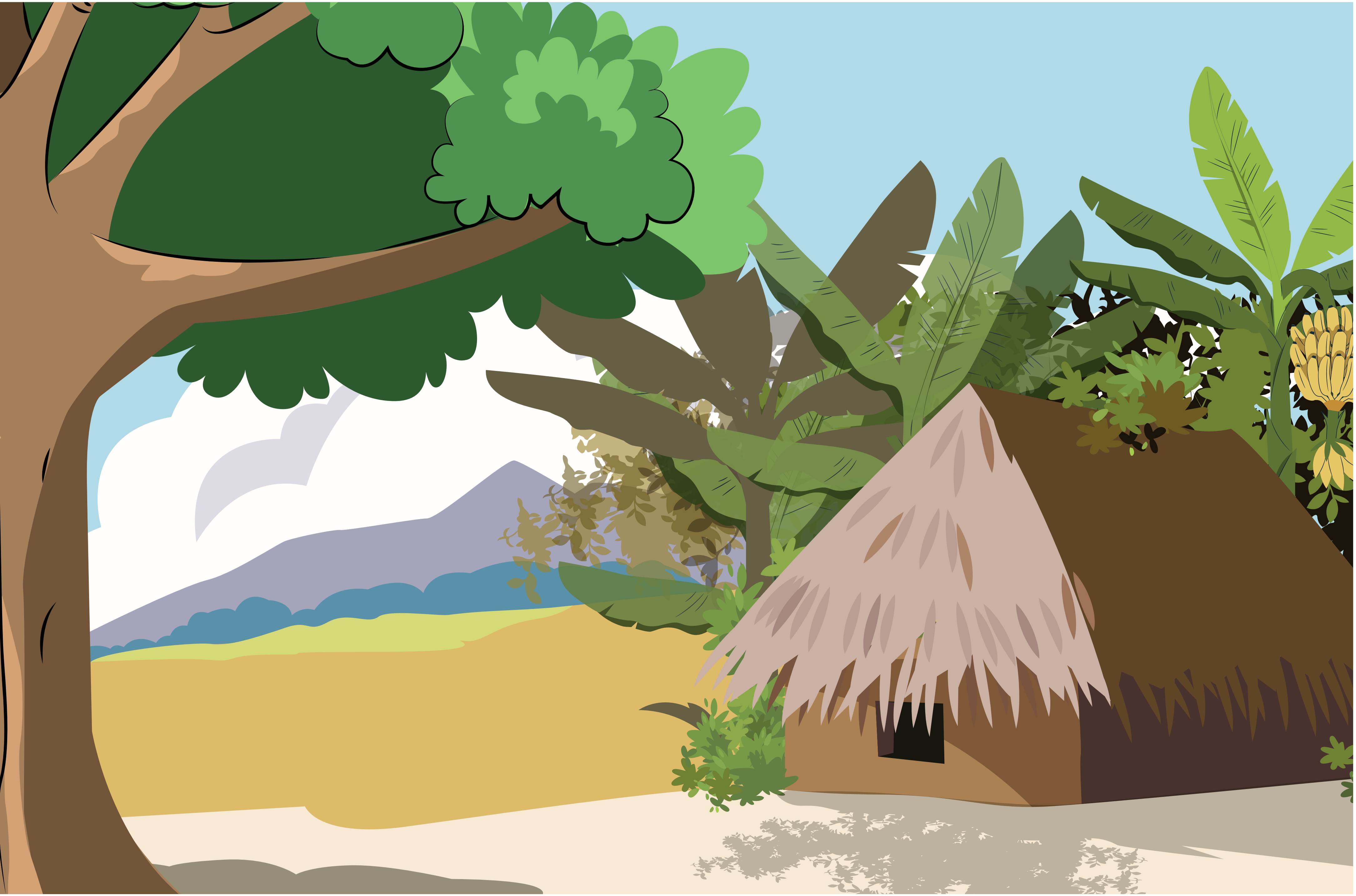

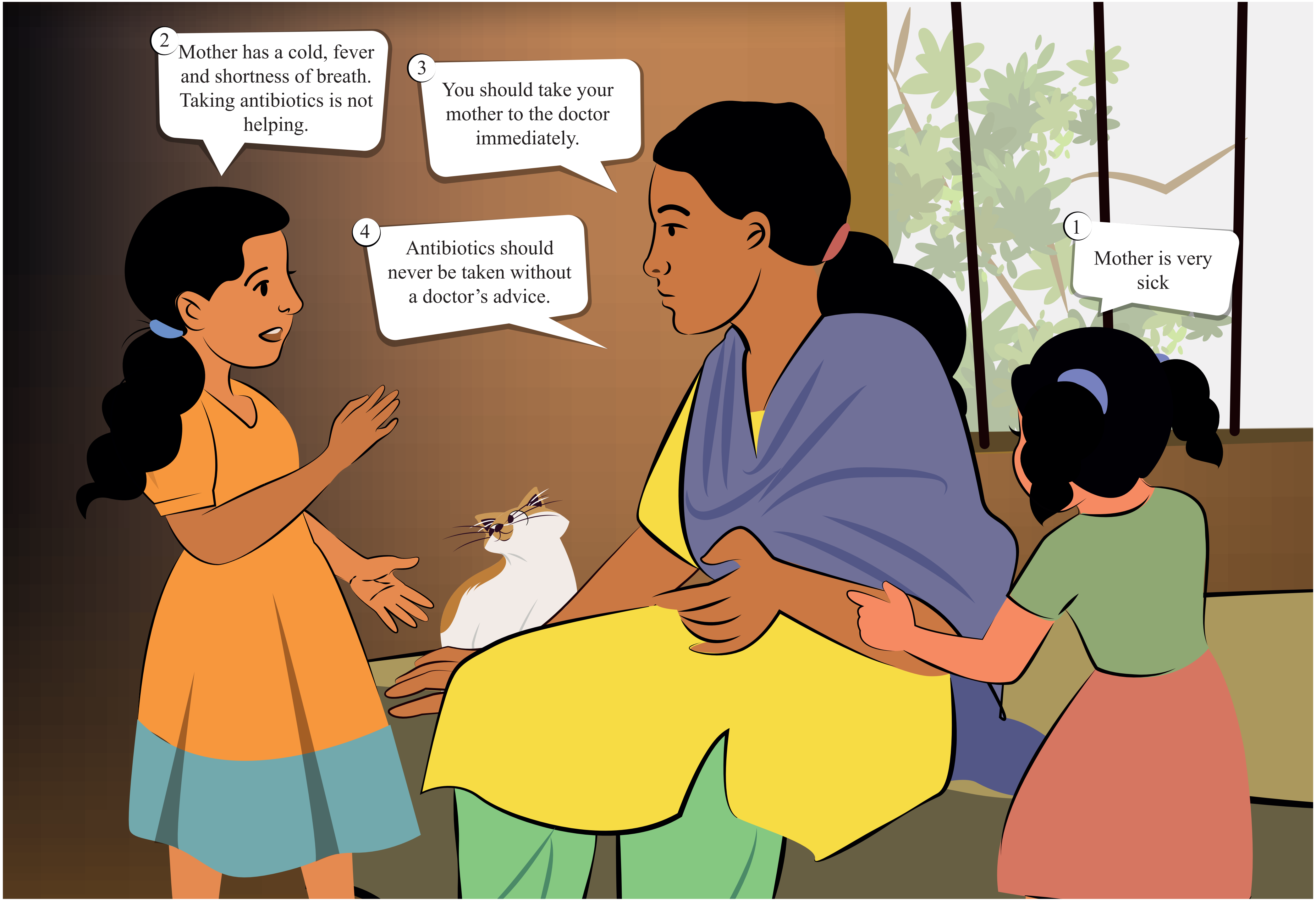
1 Mother is very sick
2 Mother has a cold, fever and shortness of breath. Taking antibiotics is not helping.
3 You should take your mother to the doctor immediately.
4 Antibiotics should never be taken without a doctor's advice.

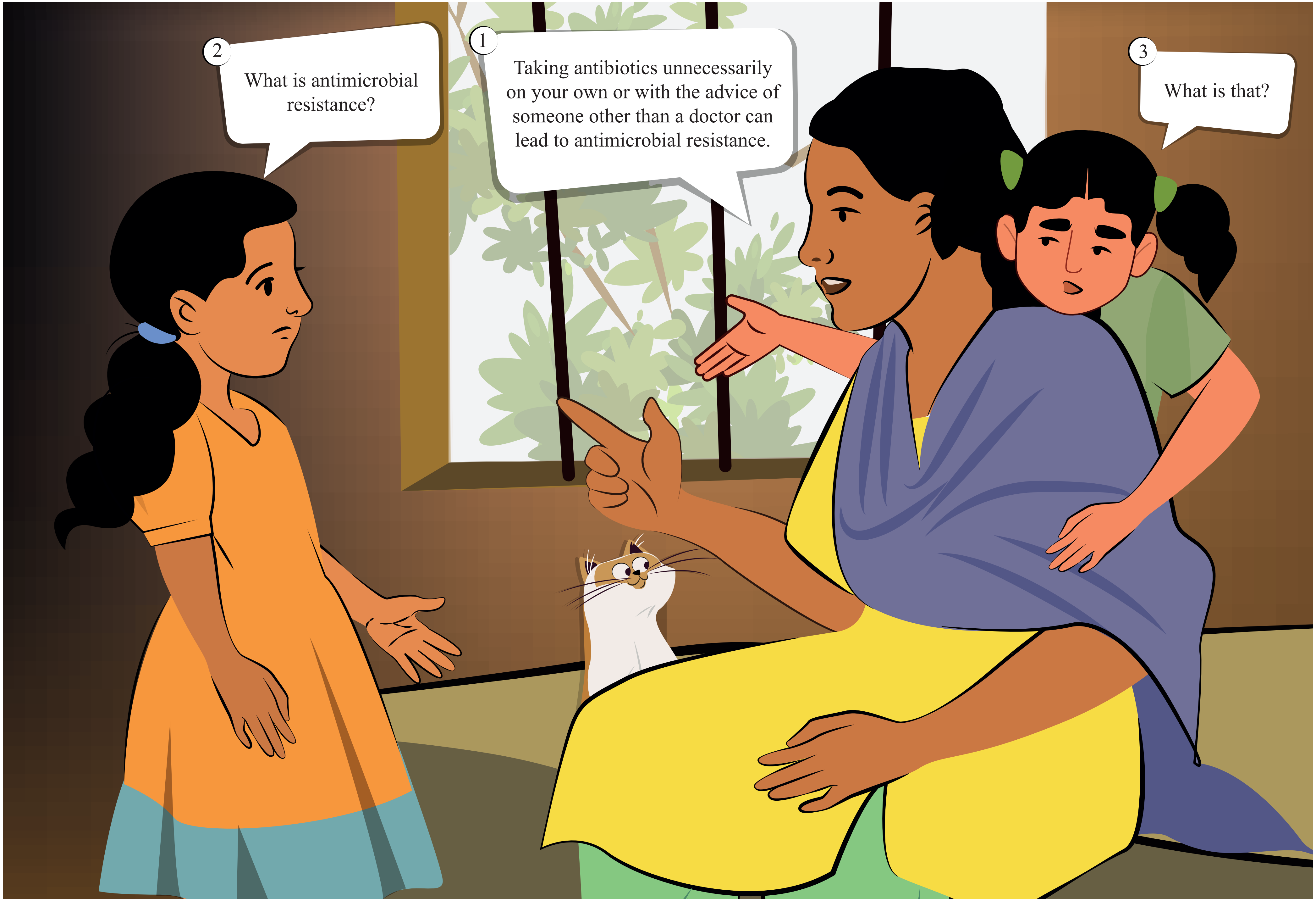
1
Taking antibiotics unnecessarily on your own or with the advice of someone other than a doctor can lead to antimicrobial resistance.
2
What is antimicrobial resistance?
3
What is that?

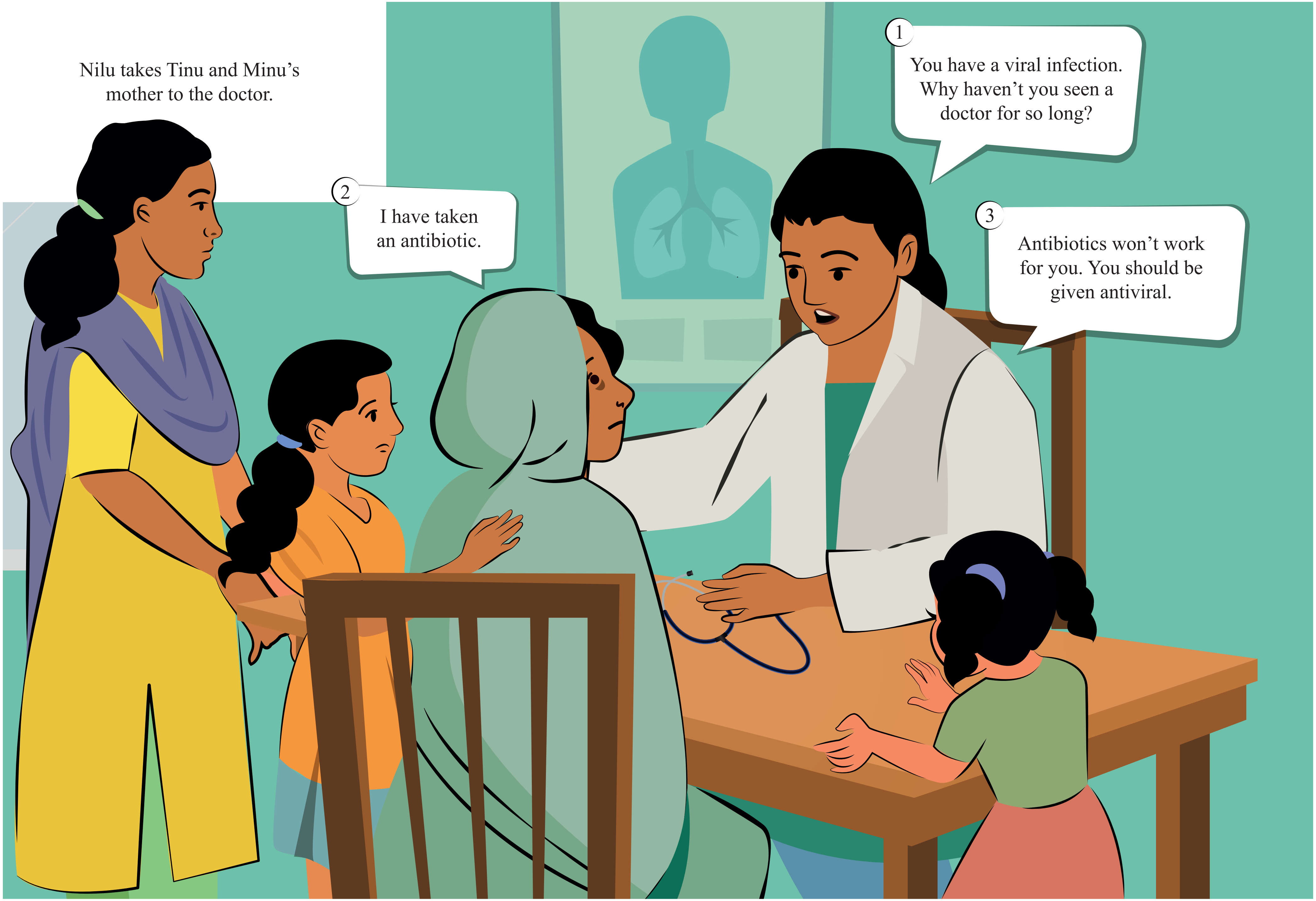
Nilu takes Tinu and Minu's mother to the doctor.
1 You have a viral infection. Why haven't you seen a doctor for so long?
2 I have taken an antibiotic.
3 Antibiotics won't work for you. You should be given antiviral.

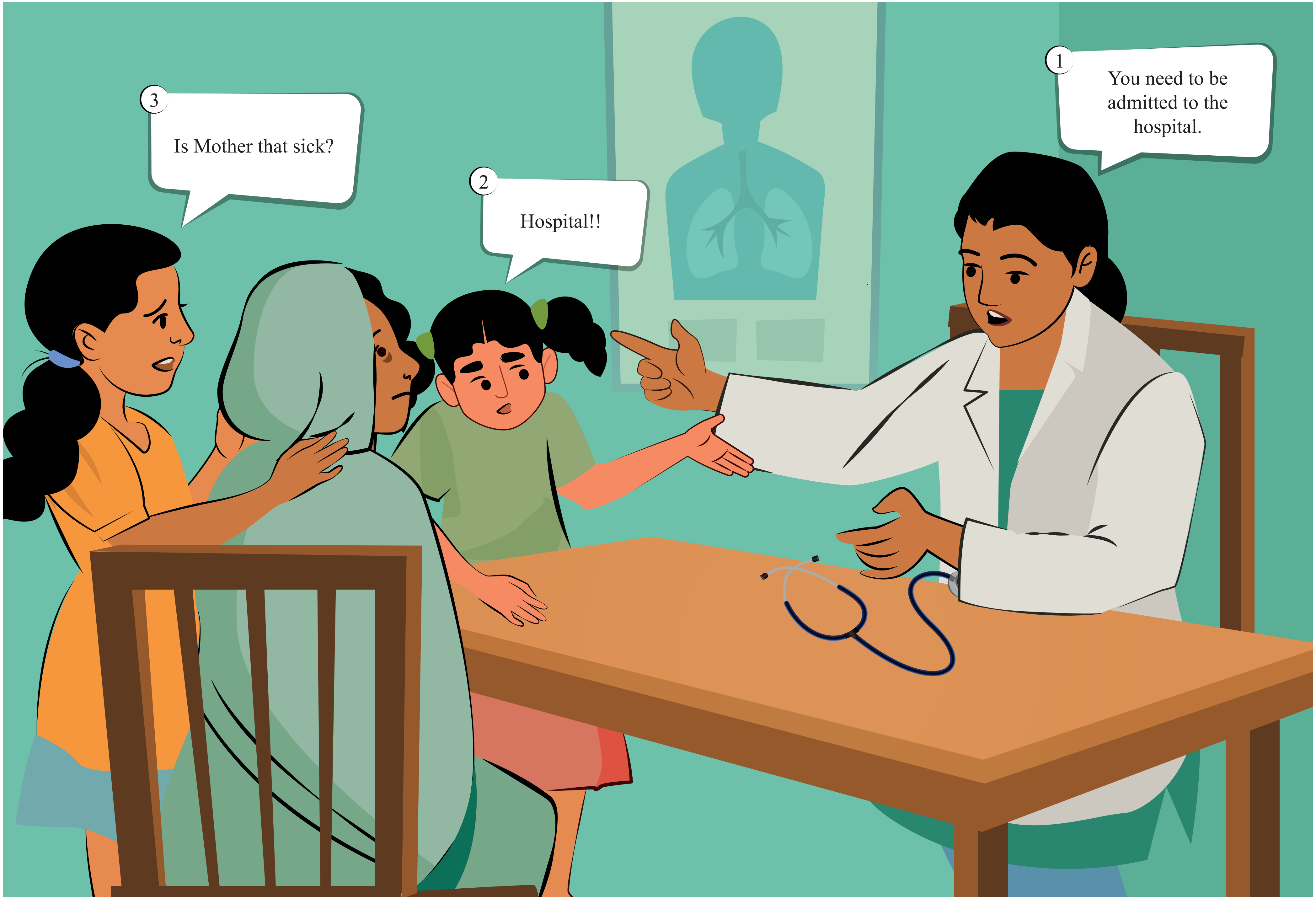
1
You need to be admitted to the hospital.
2
Hospital!!
3
Is Mother that sick?

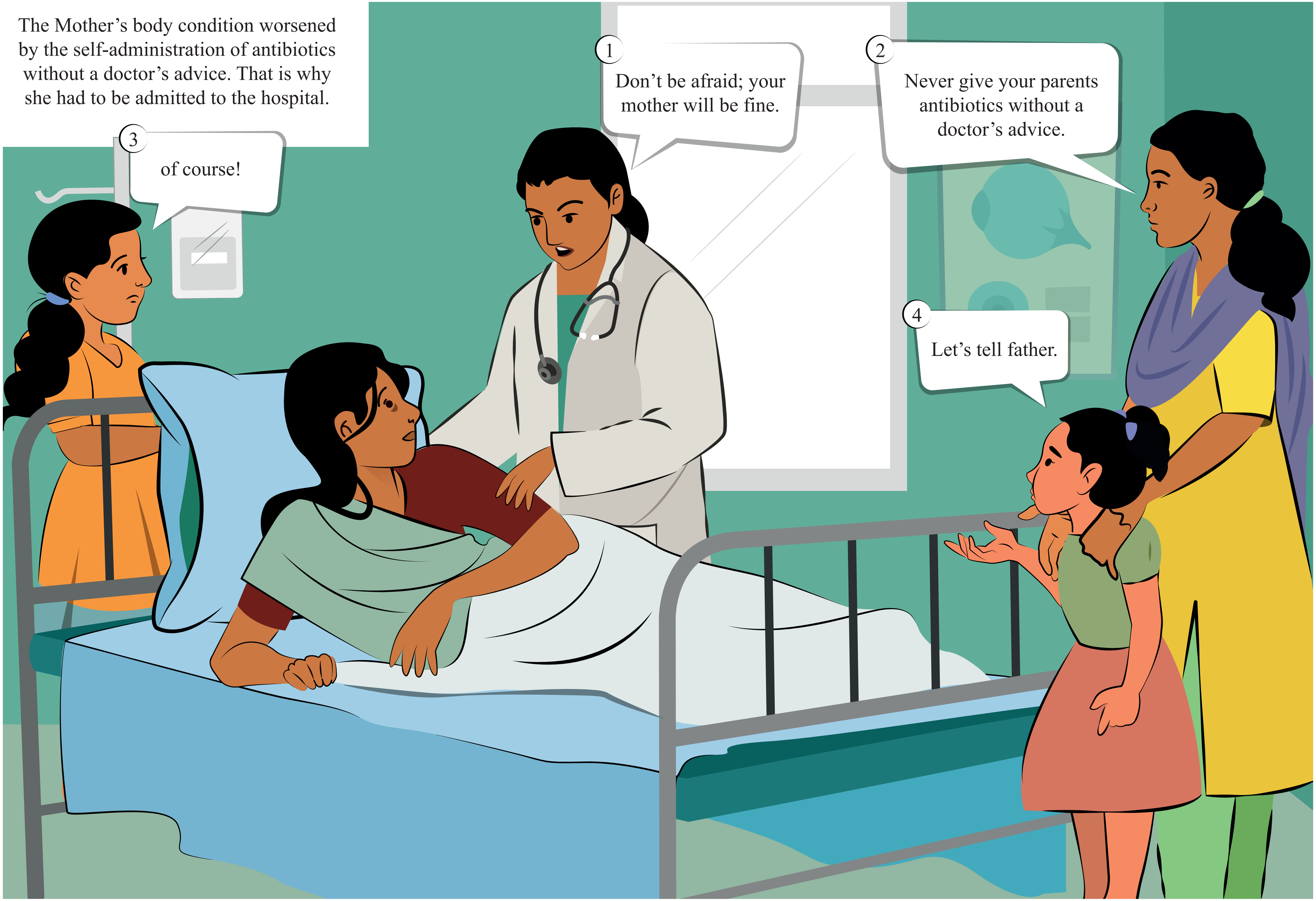
The Mother's body condition worsened by the self-administration of antibiotics without a doctor's advice. That is why she had to be admitted to the hospital.
1 Don't be afraid; your mother will be fine.
2 Never give your parents antibiotics without a doctor's advice.
3 of course!
4 Let's tell father.

The Mother is in the hospital.
She is getting better.
The viruses in the mother's body are dying. The immune system is winning.

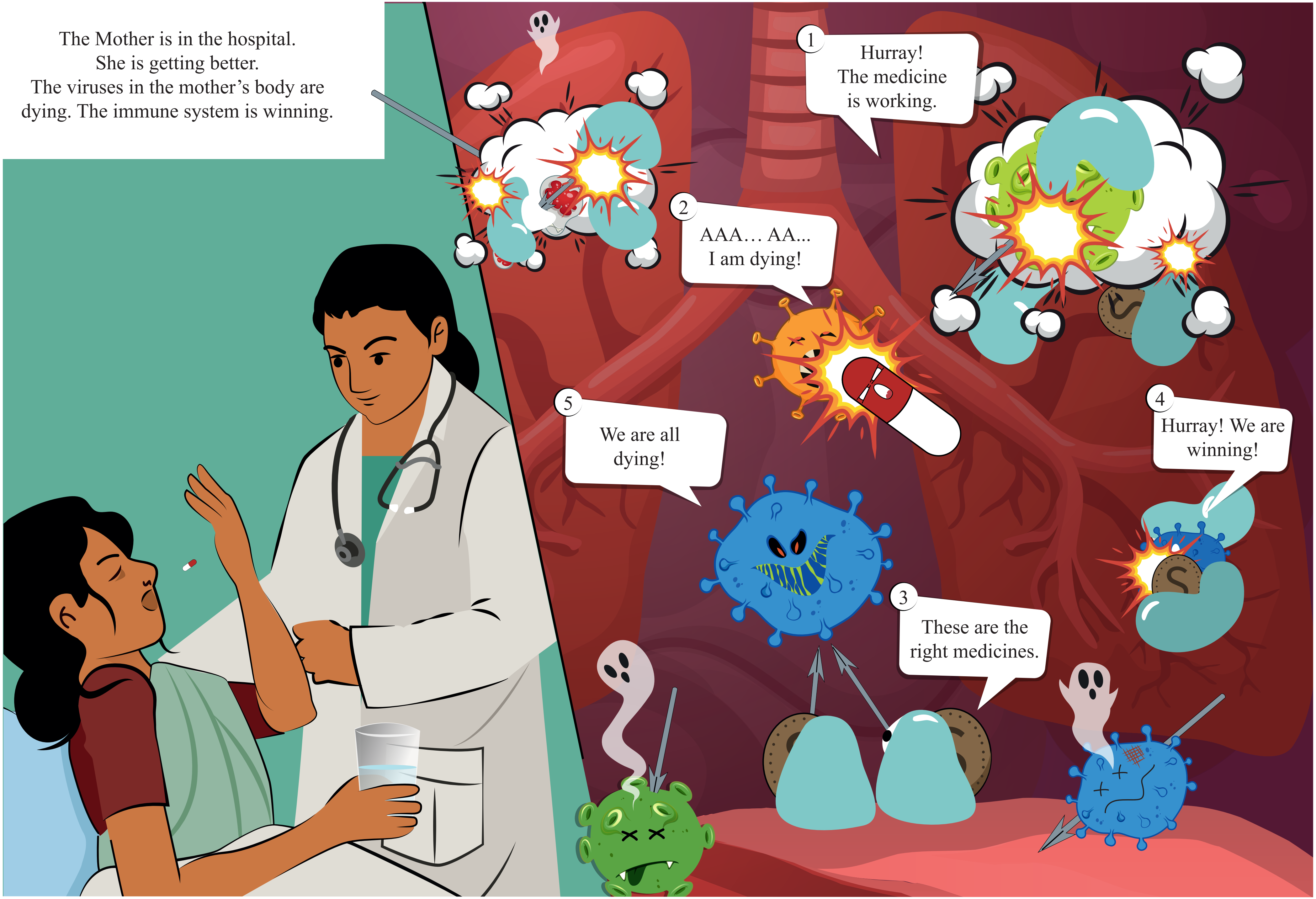

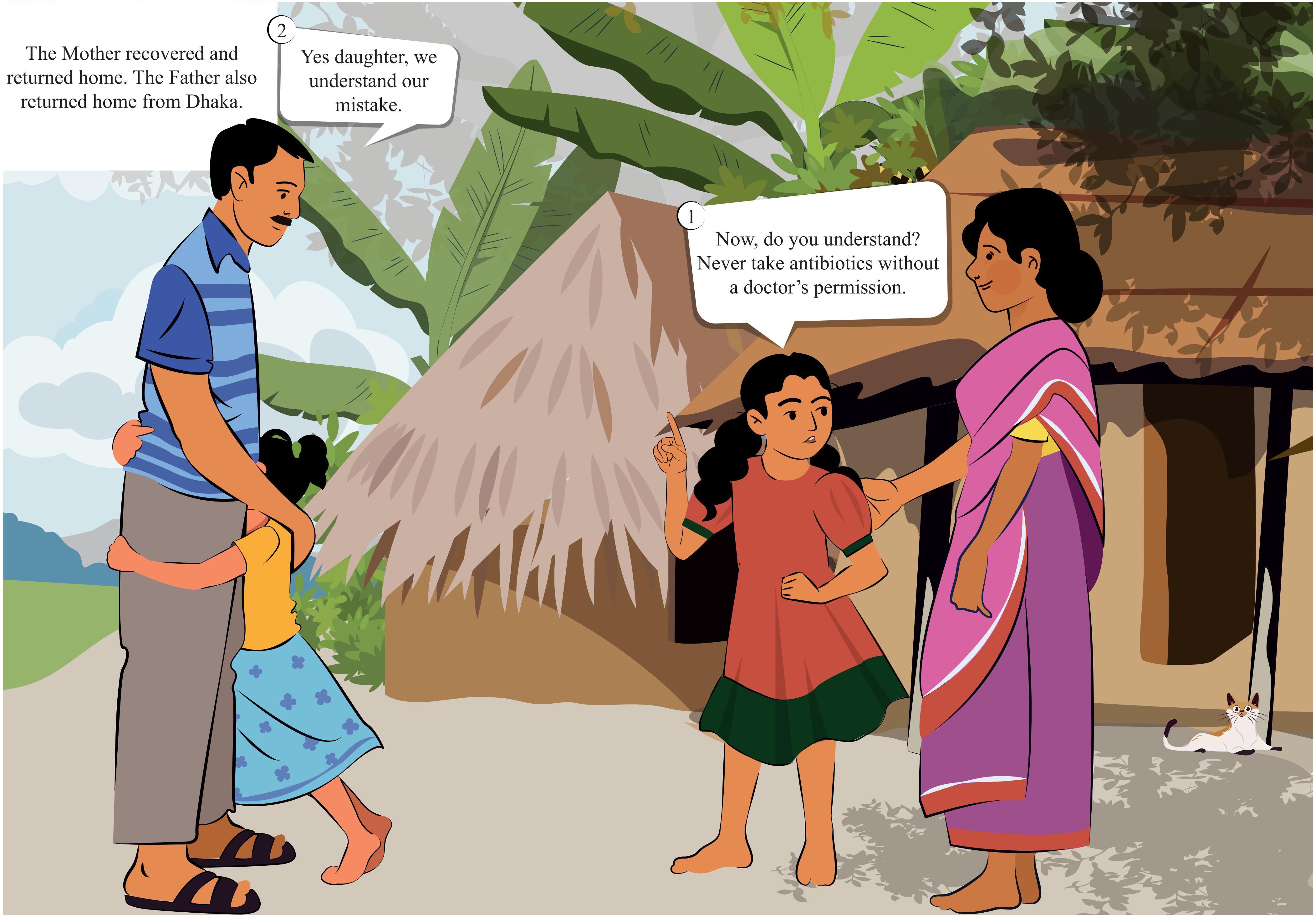
The Mother recovered and returned home. The Father also returned home from Dhaka.
1
Now, do you understand? Never take antibiotics without a doctor's permission.
2
Yes daughter, we understand our mistake.

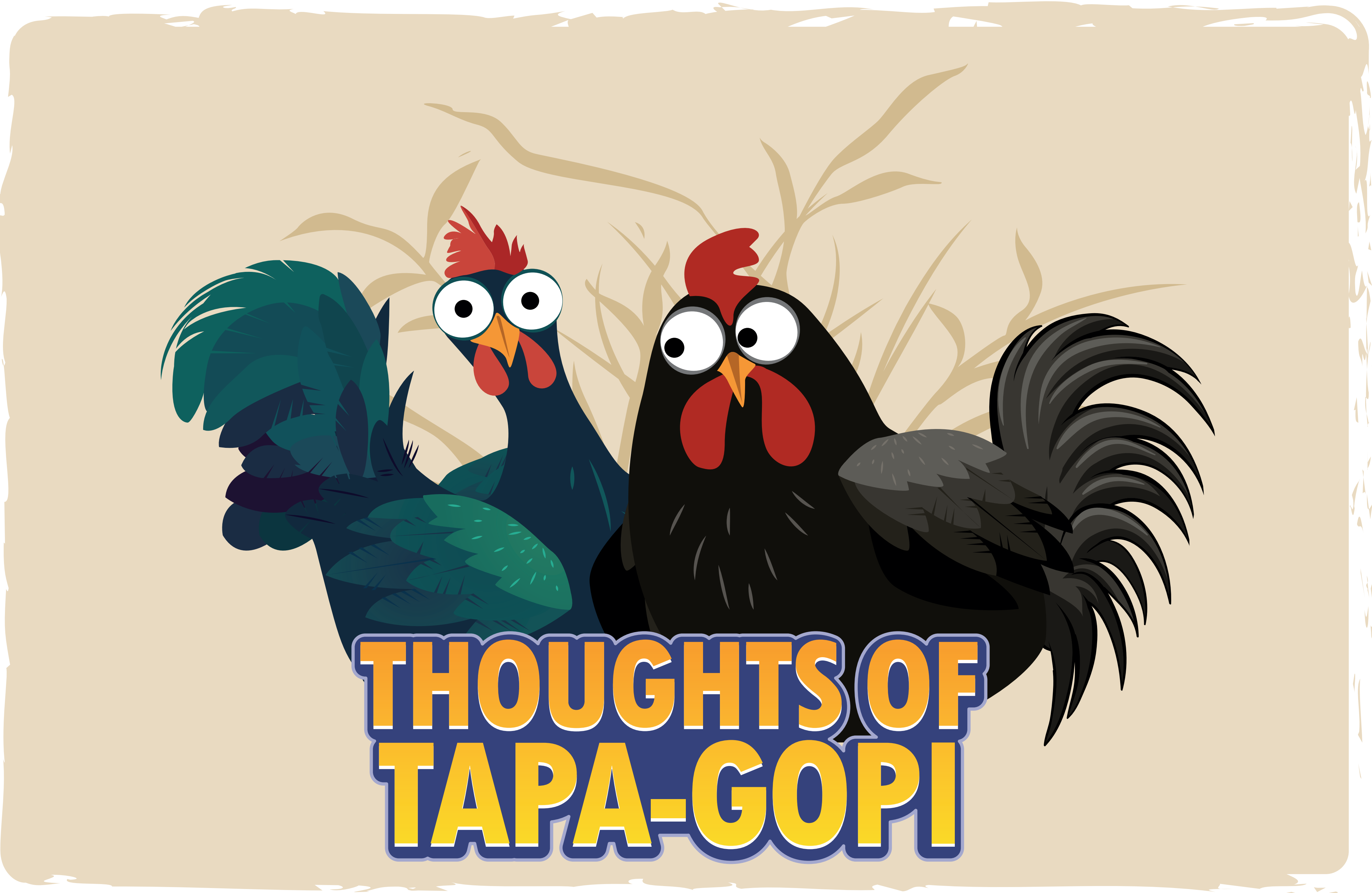
THOUGHTS OF
TAPA-GOPI

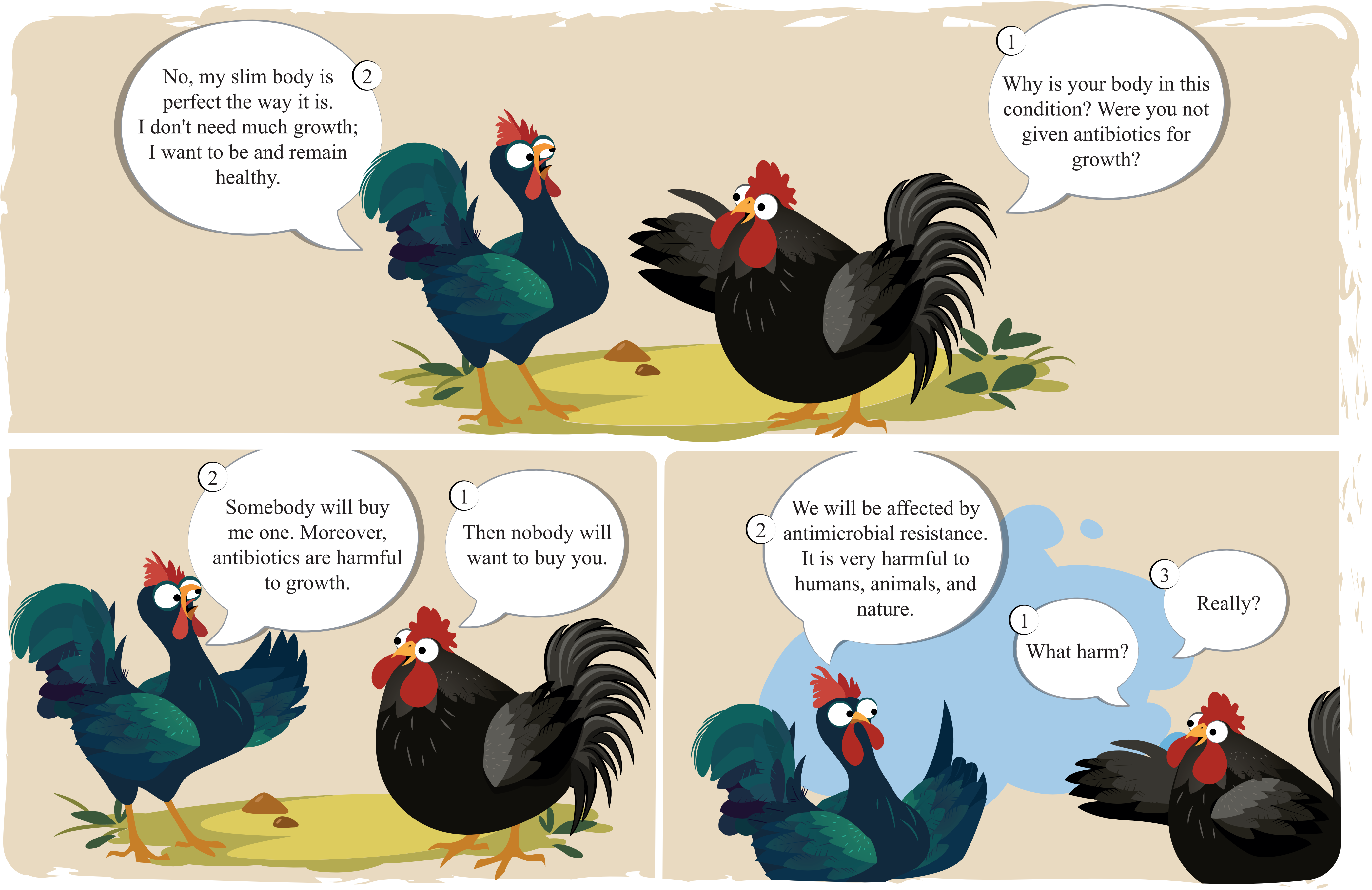
Why is your body in this condition? Were you not given antibiotics for growth?
No, my slim body is perfect the way it is. I don't need much growth; I want to be and remain healthy.
Then nobody will want to buy you.
Somebody will buy me one. Moreover, antibiotics are harmful to growth.
What harm?
We will be affected by antimicrobial resistance. It is very harmful to humans, animals, and nature.
Really?

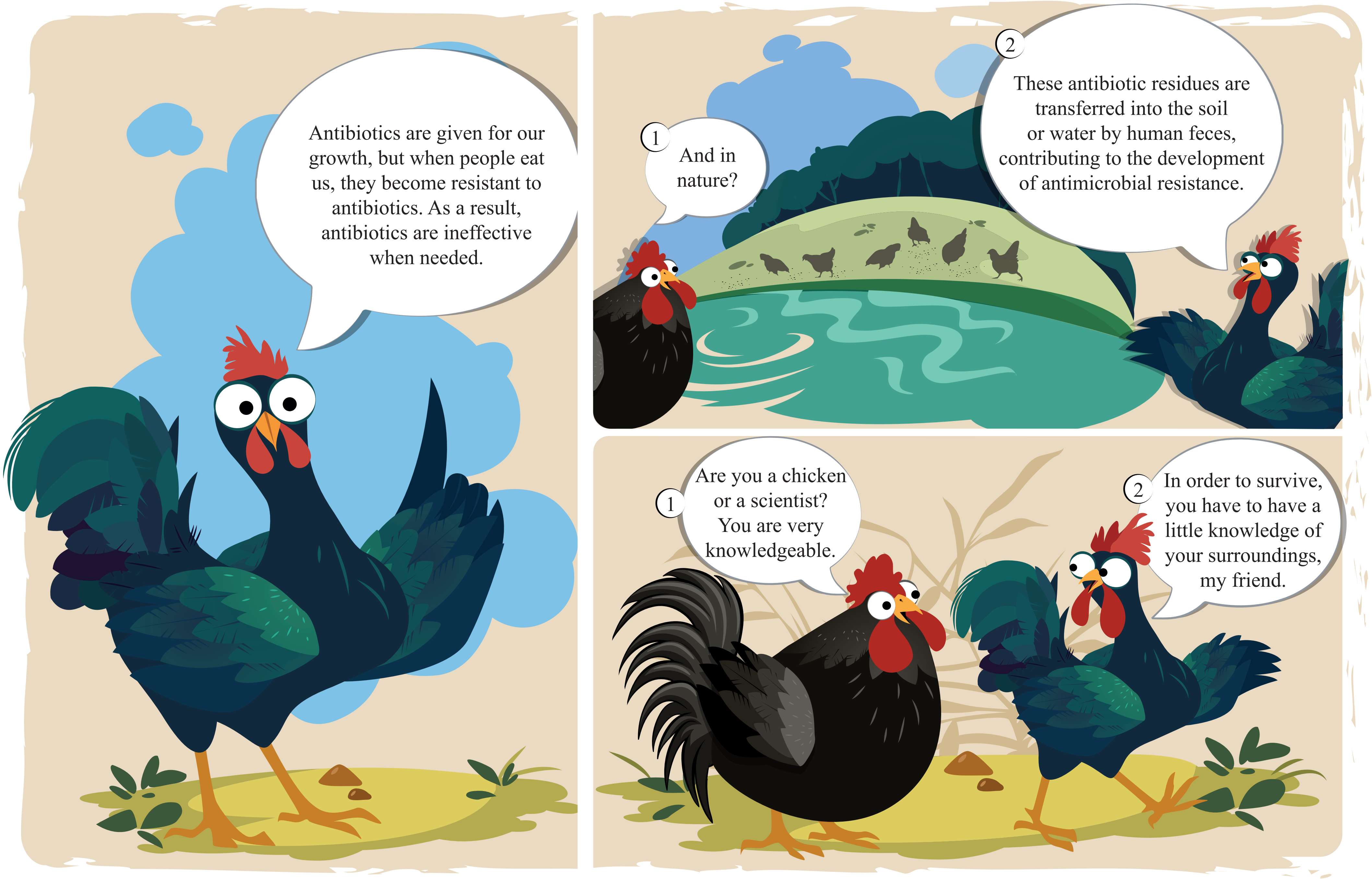
Antibiotics are given for our growth, but when people eat us, they become resistant to antibiotics. As a result, antibiotics are ineffective when needed.
1
And in nature?
2
These antibiotic residues are transferred into the soil or water by human feces, contributing to the development of antimicrobial resistance.
1
Are you a chicken or a scientist? You are very knowledgeable.
2
In order to survive, you have to have a little knowledge of your surroundings, my friend.

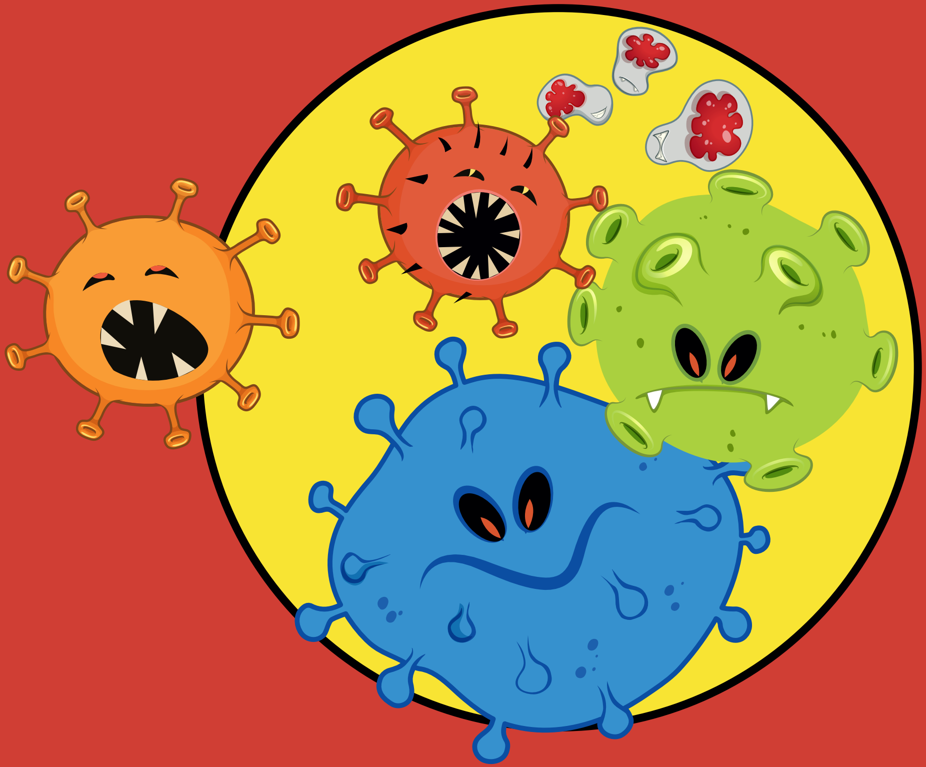

Published by:

Financial and Technical supported by:

Sweden Sverige

World Health Organization Bangladesh
