# Supplementary material for: Assessing the Impact of Antimicrobial Resistance Awareness Interventions Among Schoolchildren in Bangladesh
Source: Antibiotics (Basel). 2025 Sep 29;14(10):979. doi: 10.3390/antibiotics14100979 (PMC12561141; doi:10.3390/antibiotics14100979)

ANTIBIOTICS COLORING BOOK

Invention of Penicillin

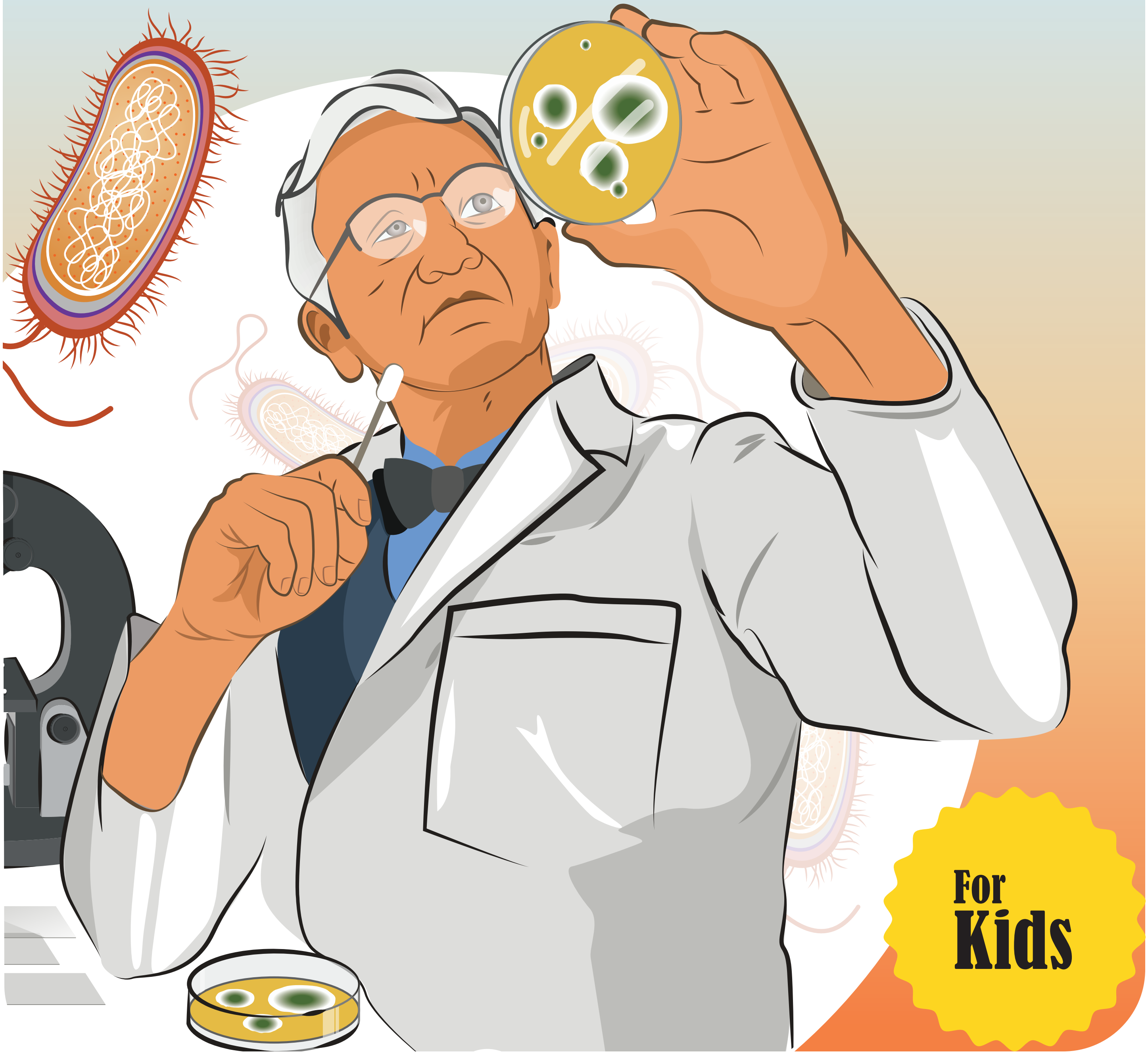

www.dgda.gov.bd

For Kids

Coloring Book

Invention of Penicillin

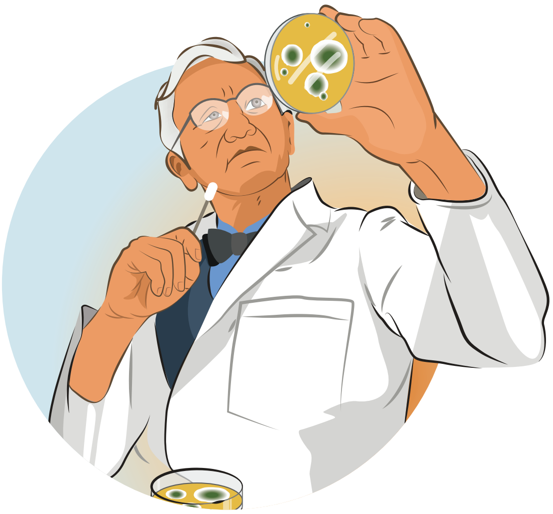

EDITORIAL BOARD

MAJOR GENERAL MOHAMMAD YOUSUF
DIRECTOR GENERAL, DIRECTORATE GENERAL OF DRUG ADMINISTRATION
MR. MD. MOSTAFIZUR RAHMAN
DIRECTOR, DIRECTORATE GENERAL OF DRUG ADMINISTRATION
MR. MD. ABDUL BARI
ASSISTANT DIRECTOR, DIRECTORATE GENERAL OF DRUG ADMINISTRATION
MS. S. M. SABRINA YESMIN
ASSISTANT DIRECTOR, DIRECTORATE GENERAL OF DRUG ADMINISTRATION
MR. ATM GOLAM KIBRIA KHAN
ASSISTANT DIRECTOR, DIRECTORATE GENERAL OF DRUG ADMINISTRATION
MR. MD. BASHARAF HOSSAIN
INSPECTOR OF DRUGS, DIRECTORATE GENERAL OF DRUG ADMINISTRATION
MS. UMME HABIBA
NATIONAL CONSULTANT-AMR,DGDA, WHO-BANGLADESH
MR. PARITOSH CHAKMA
NATIONAL CONSULTANT- ESSENTIAL DRUGS AND MEDICINES
WHO- BANGLADESH

COVER AND GRAPHICS

MS. S. M. SHANZIDA YEASMIN
DHAKA, BANGLADESH

FIRST RELEASE . JULY 2023

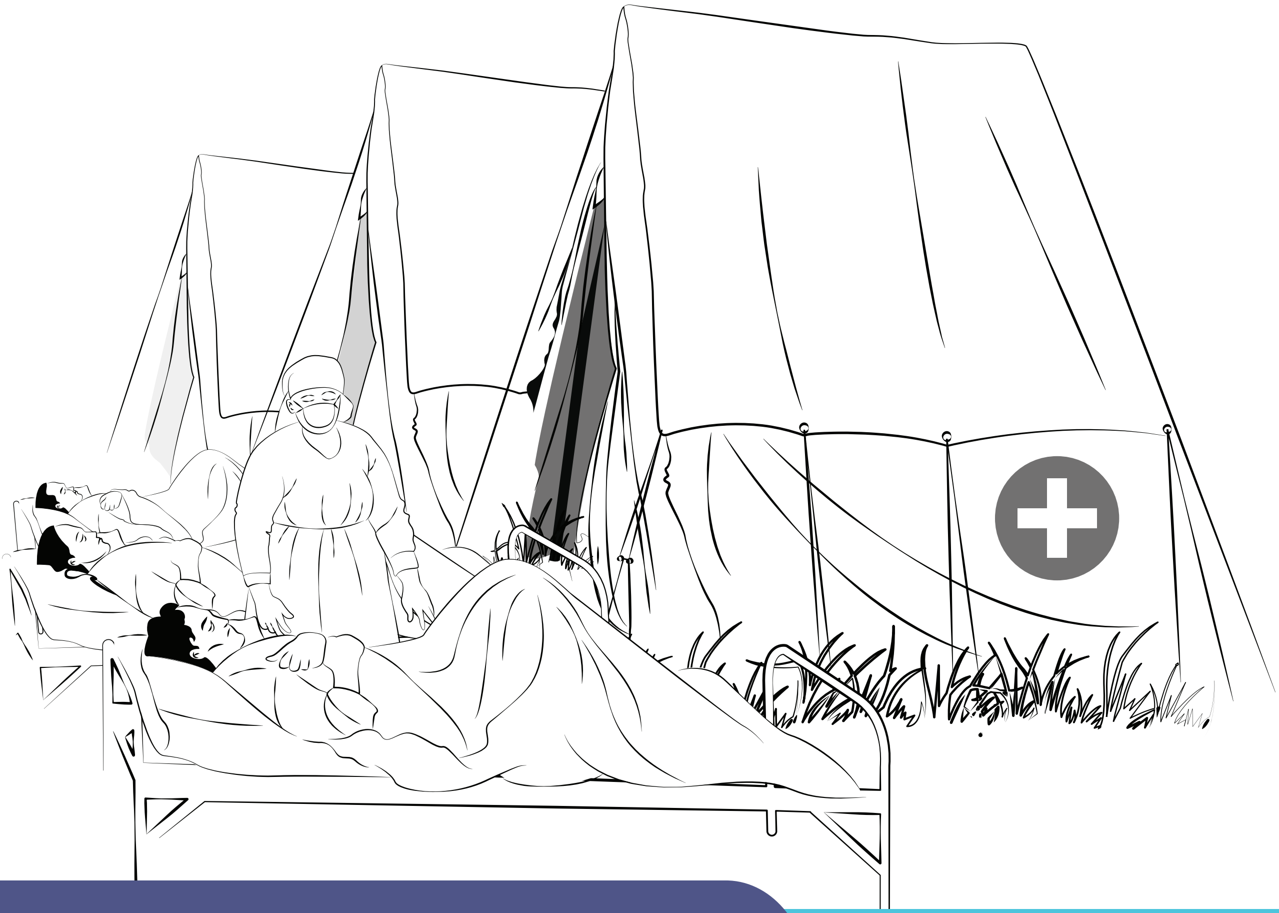

The Amazing Discovery of Penicillin

1

Once upon a time, long ago, there were no antibiotics in the world to fight dangerous infections. People would get sick, and some even lost their lives because there was no medicine to help them get better.

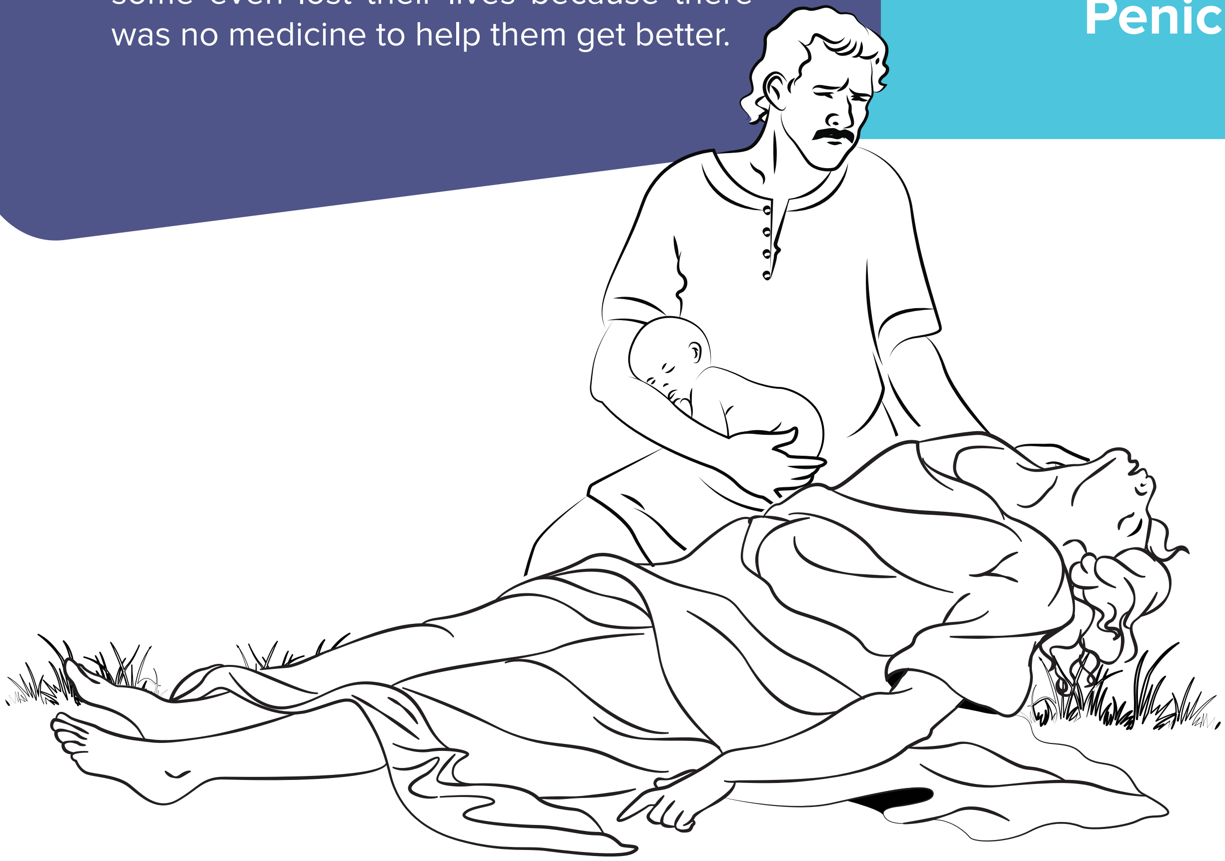

2

But then, something incredible happened! A bright scientist Dr. Alexander Fleming, loved to study tiny bacteria that you couldn't see without a special tool called a microscope. He worked at Saint Mary's Hospital in London, where he researched different types of bacteria. Among them one is called "Staphylococci."

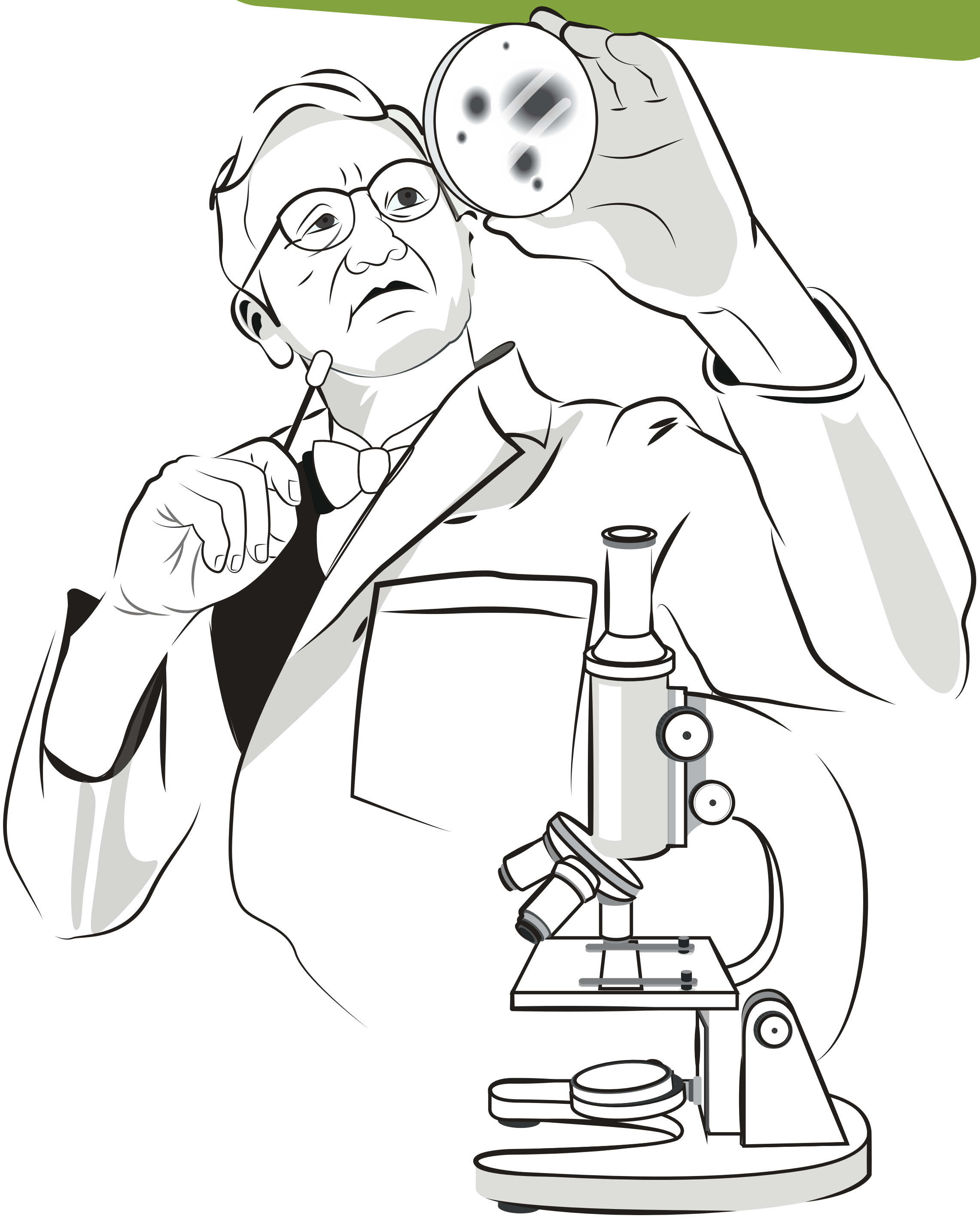

3

One day in 1928, Dr. Fleming was doing important experiments with bacteria. He carefully placed the bacteria on a dish called a petri dish and went for his summer vacation.

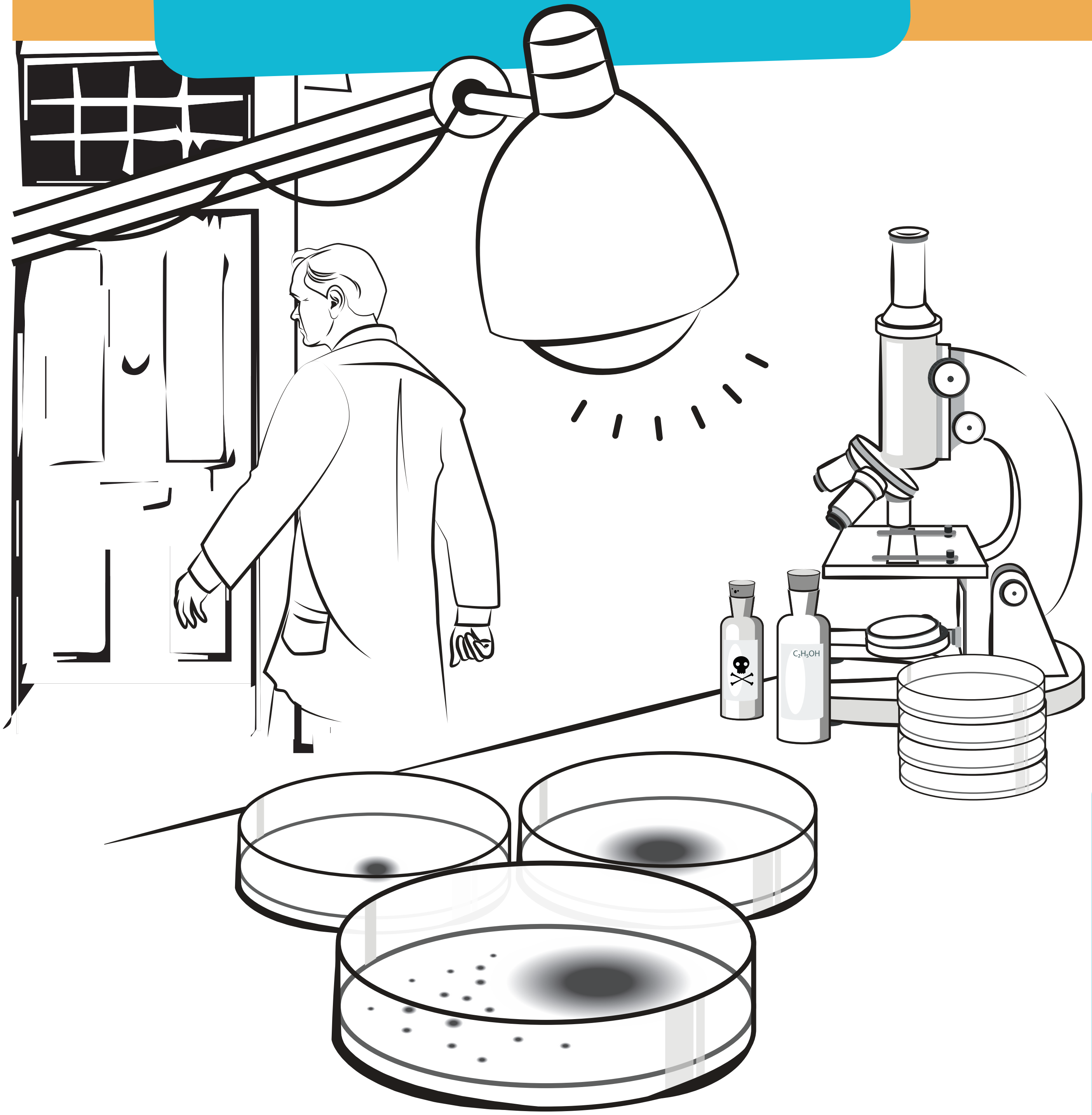

4

When he returned, he found his lab was messy, and he was worried that the bacteria had been ruined.

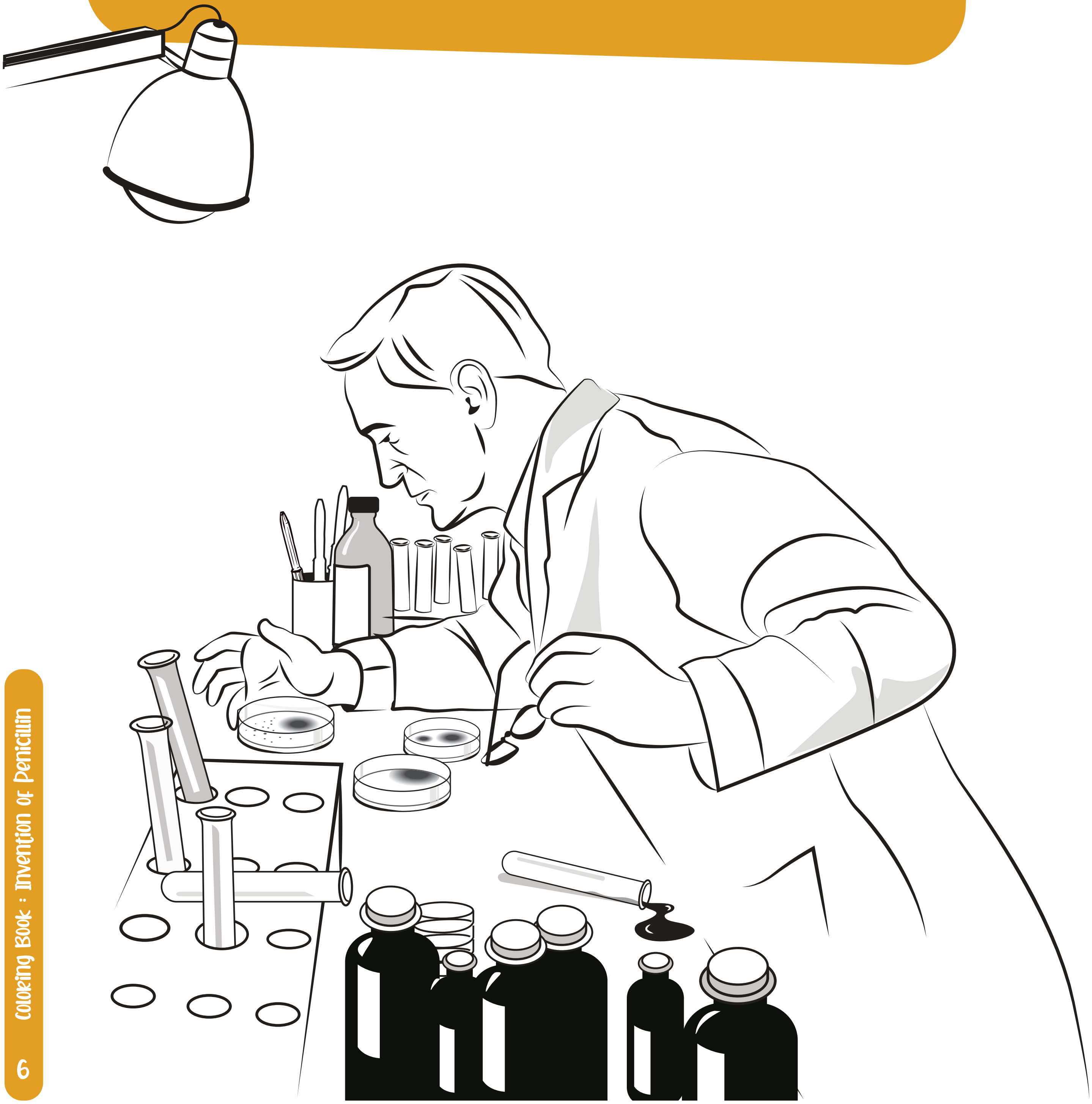

5

But something surprising was waiting for him. He discovered a special fungus had grown on the petri dish and killed the bacteria. He decided to call this powerful fungus "Penicillin."

Dr. Fleming realized that Penicillin could be the key to helping people fight against infections and recover. This incredible discovery meant that Penicillin could stop bacteria from growing and even kill them, just like a miraculous drug fighting the harmful germs inside our bodies. It was the world's first-ever antibiotic!

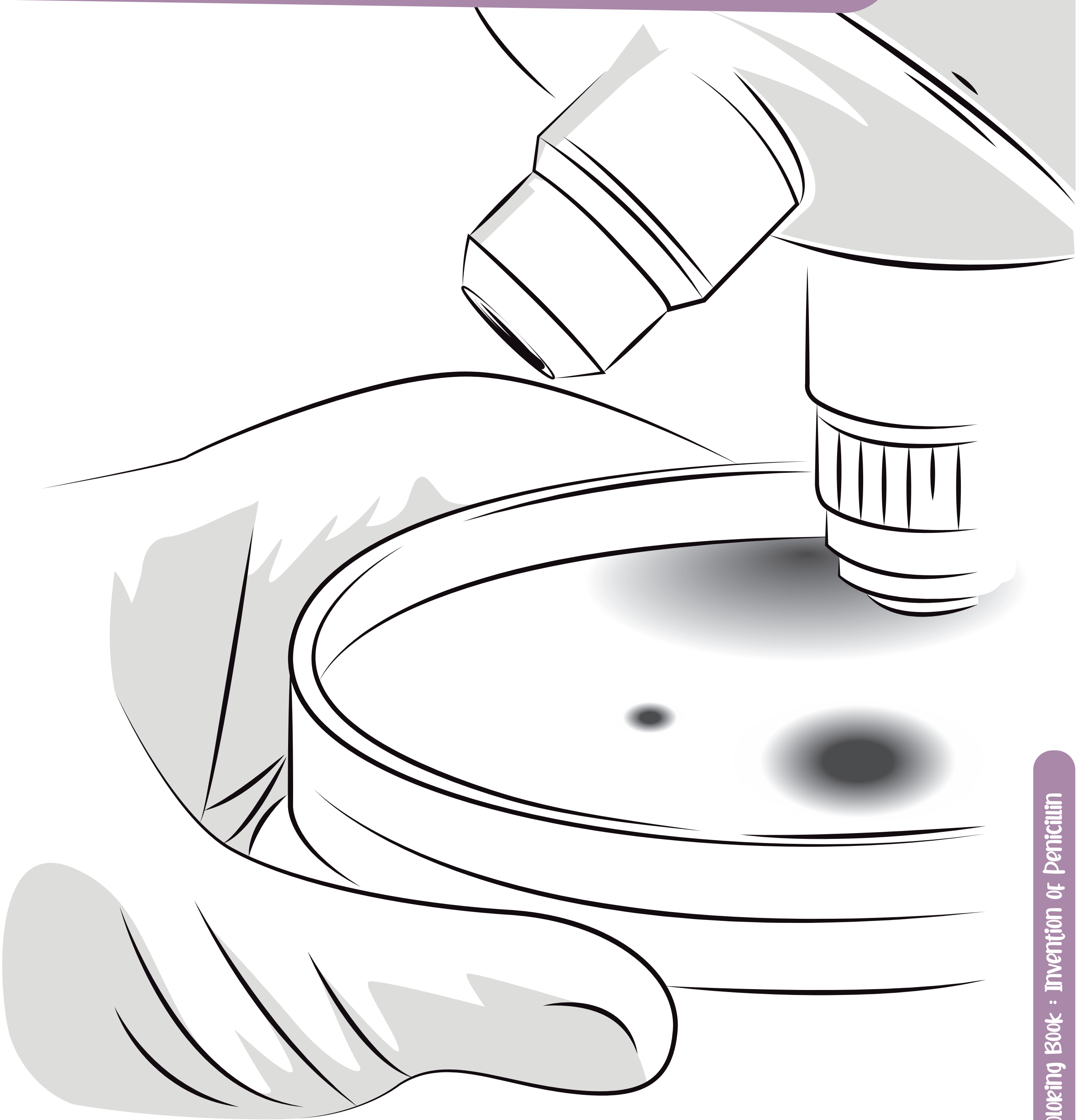

6

In 1942, a seriously ill patient with a severe blood infection was treated with Penicillin as a medicine. Everyone thought the patient might not survive, but they were mistaken. With the miraculous powers of Penicillin, the patient improved and survived!

And that's how the brave scientist, Dr. Alexander Fleming, invented the remarkable Penicillin, which became a miraculous medicine, saving countless lives from dangerous infections. The world became a safer place, all thanks to the power of science and discovery!

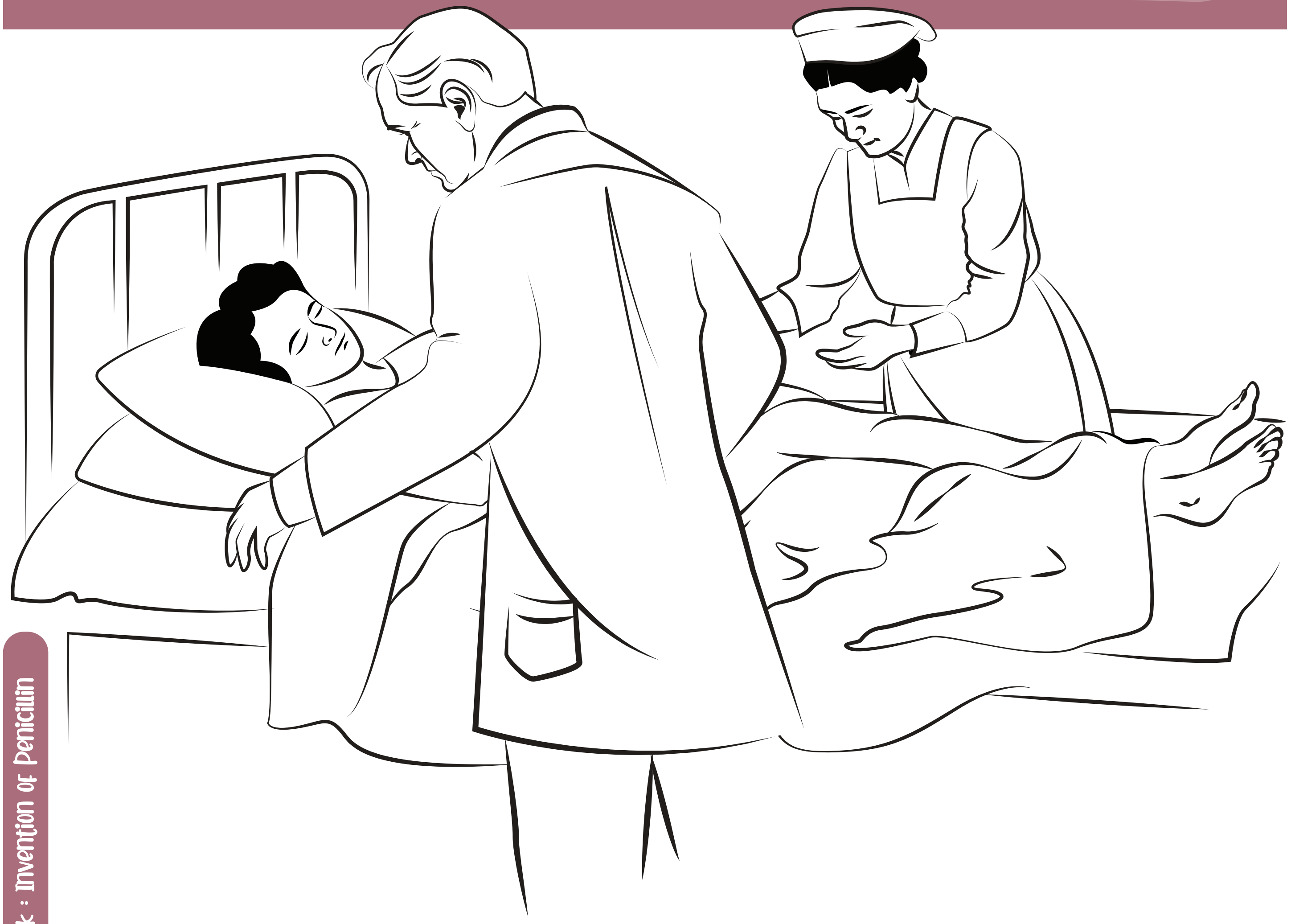

7

Bacterial infections have been successfully treated for many years since Alexander Fleming's discovery of antibiotics. Antibiotics were considered a miracle medicine in the world at that time. Later, over time, due to the misuse of antibiotics, they become ineffective in treating bacterial infections and the bacteria destroy the effectiveness of the antibiotics. This results in the onset of antimicrobial resistance.

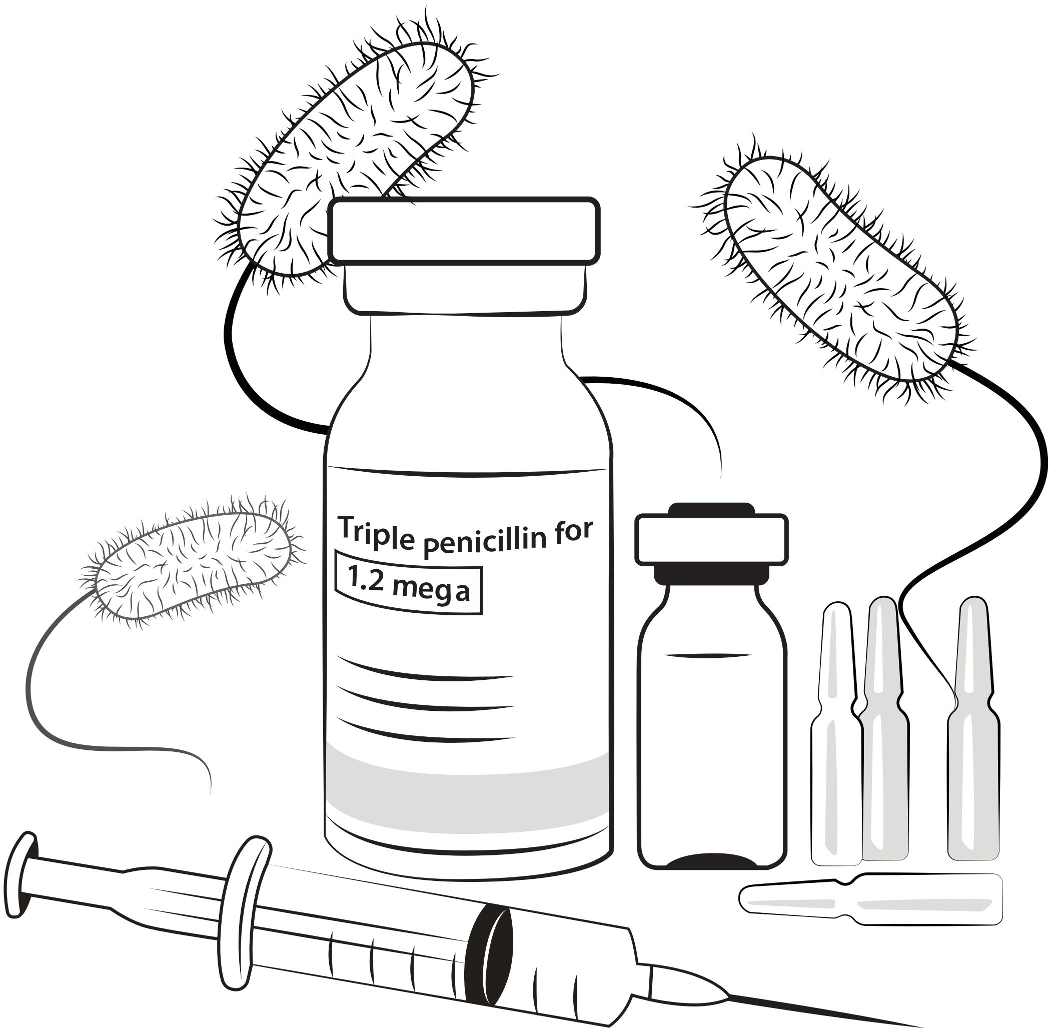

8

When microorganisms (bacteria, virus, fungus, parasite) become resistant, this resistance can spread from one organism to another. For example, corona virus could spread from one person to another. Just like that, microorganisms can also spread from one person to another, even it can spread from animal to human. Because of the misuse of antimicrobial medicines in animals, antimicrobial resistance is also spreading among humans.

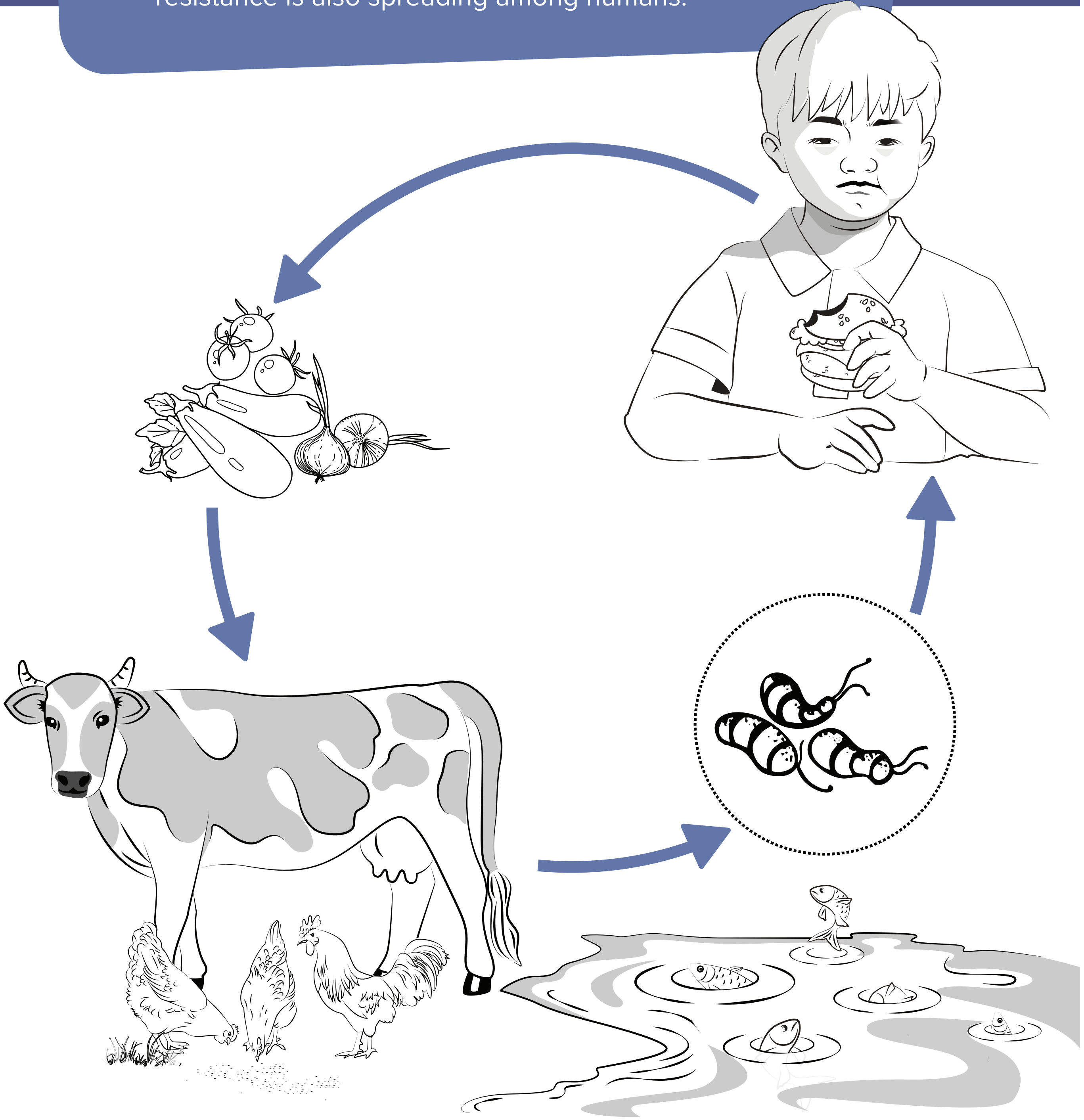

9

Today, by our own abuse, we are ruining this humanitarian invention of Alexander Fleming's. We often take antibiotics on our own accord or on the advice of relatives without consulting a doctor when we get a common cold, fever or any other disease. We forget that these antibiotics are only effective against bacterial infections. As Corona is a viral infection, antibiotics do not work in this case. We must remember that antimicrobial drugs should never be taken without a doctor's advice.

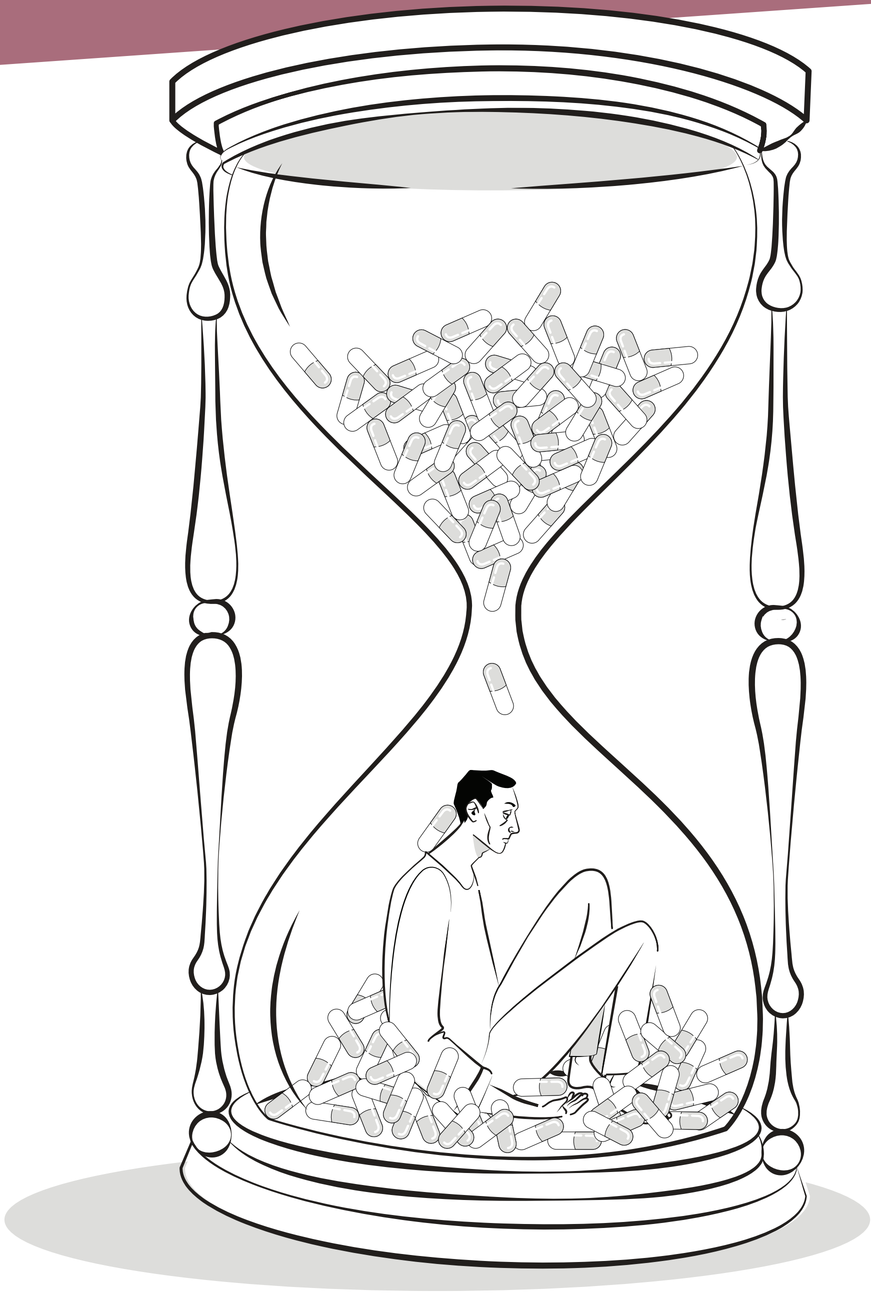

DESCRIPTION

Microorganism

A microorganism is a type of microscopic organism that cannot be seen with the naked eye, but can only be seen with the help of a microscope.
For example, bacteria, viruses, fungi, parasites, etc.

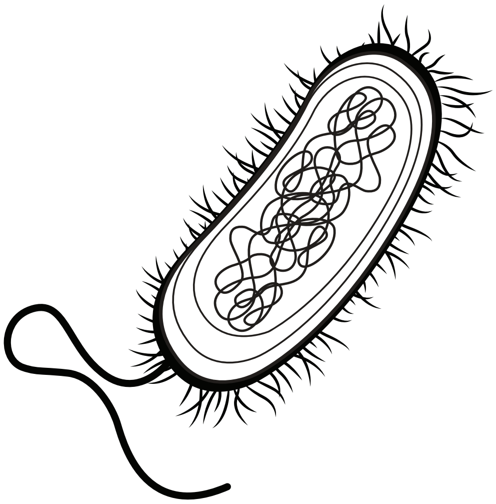

Bacteria

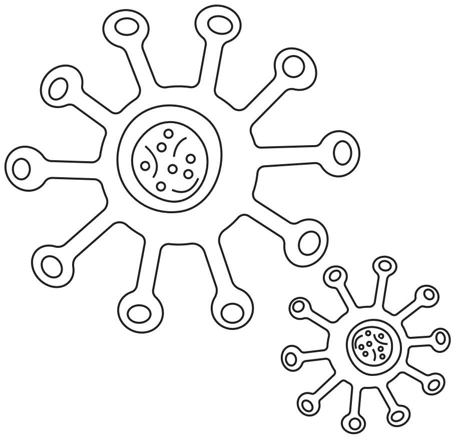

Virus

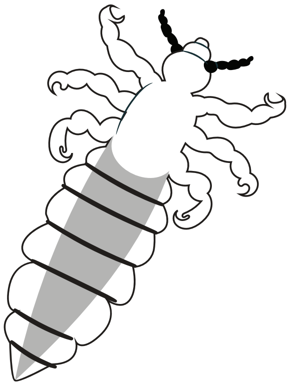

Parasite

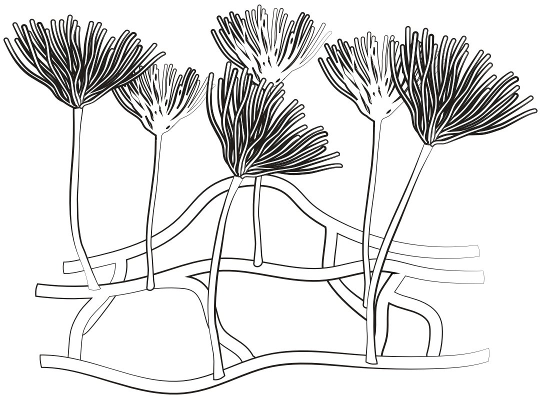

Fungi

DESCRIPTION

Antimicrobial drug

A drug used to treat a microbial infection.

"Antimicrobial" is a general term that refers to a group of drugs that includes antibiotics, antifungals, antiprotozoals, and antivirals.

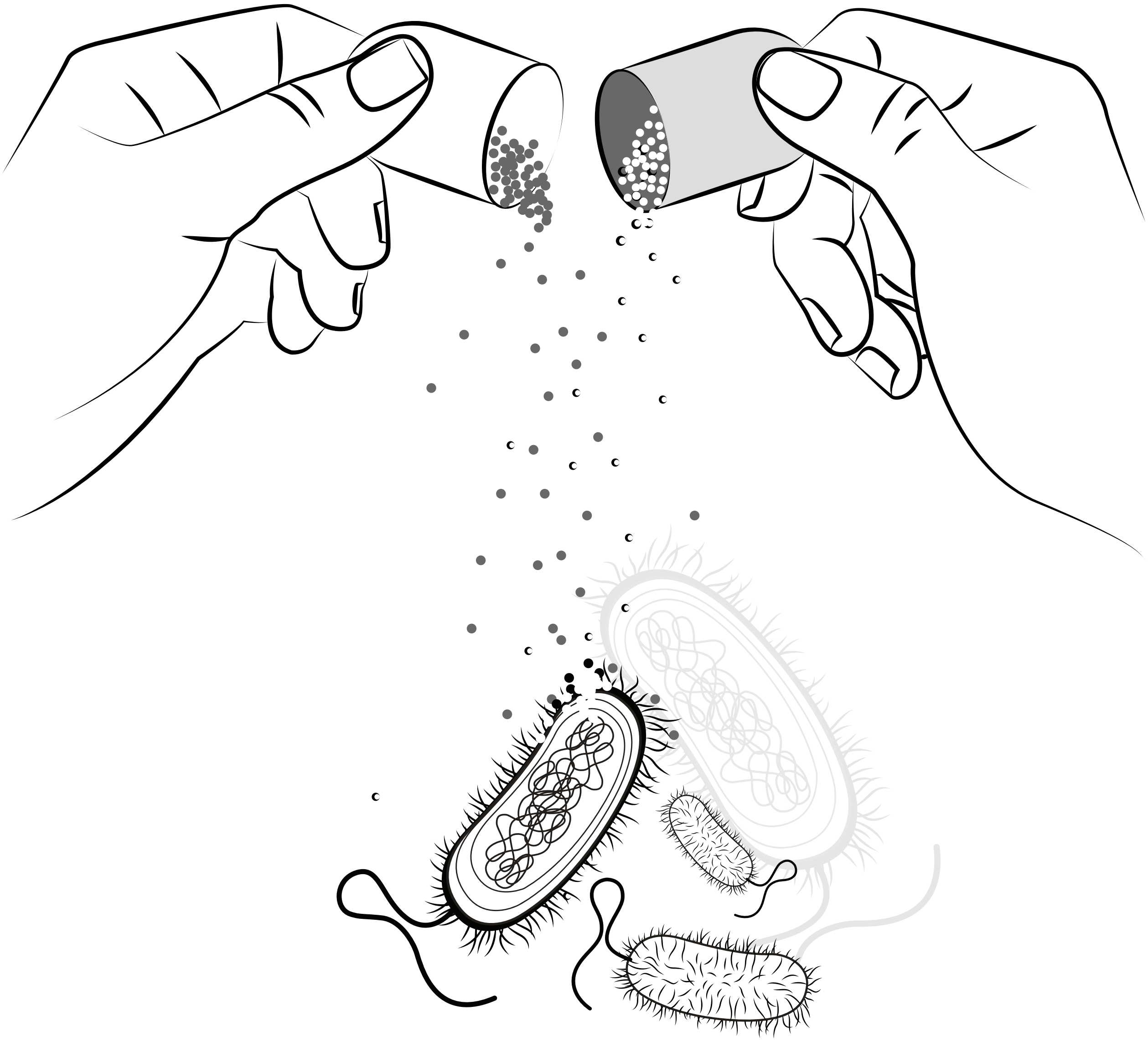

DESCRIPTION

Antimicrobial Resistance (AMR)

Antimicrobial Resistance (AMR) occurs when bacteria, viruses, fungi and parasites change over time and no longer respond to medicines making infections harder to treat and increasing the risk of disease spread, severe illness and death.

As a result of drug resistance, antibiotics and other antimicrobial medicines become ineffective and infections become increasingly difficult or impossible to treat.

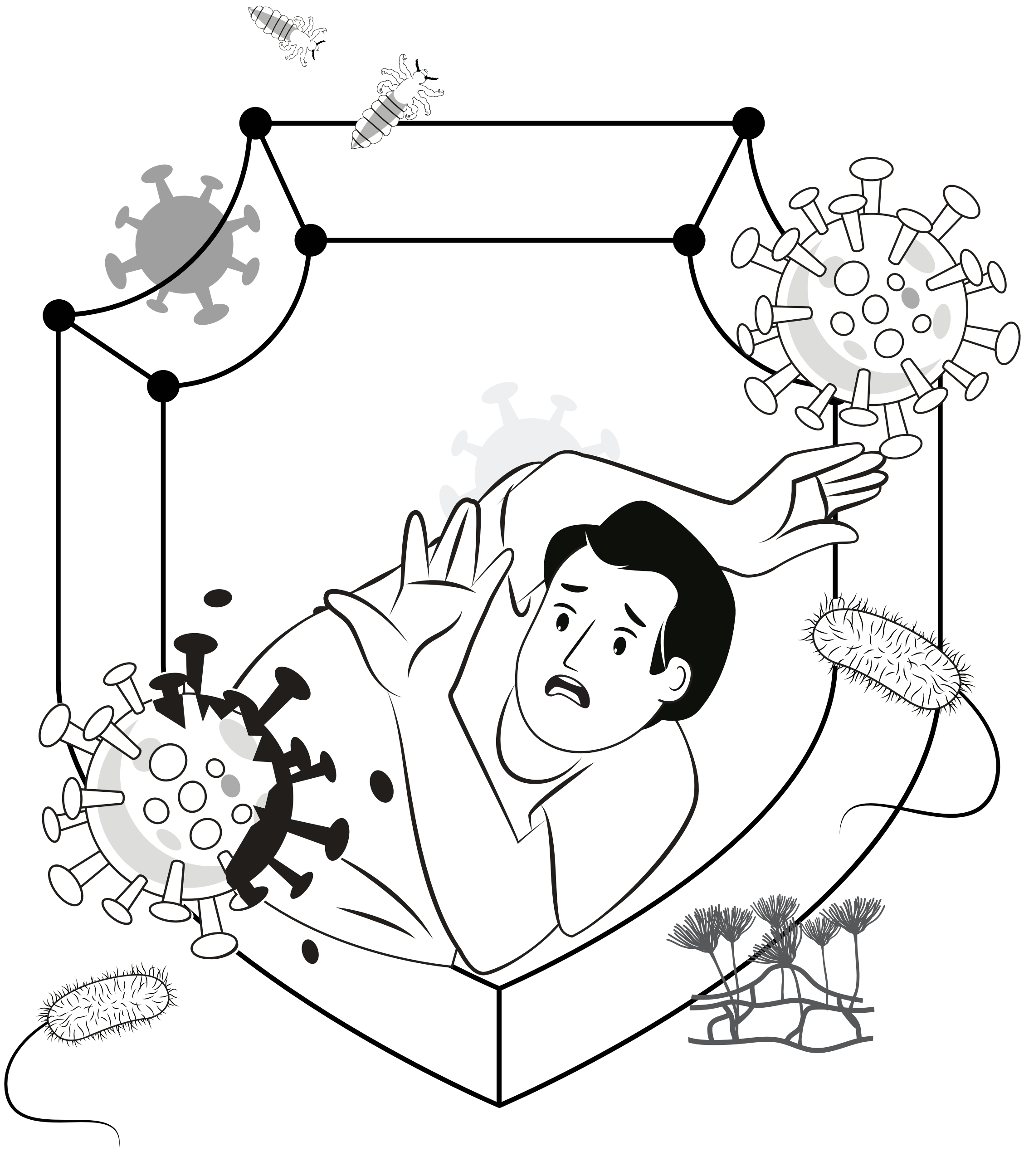

Notes

tic-tac-toe

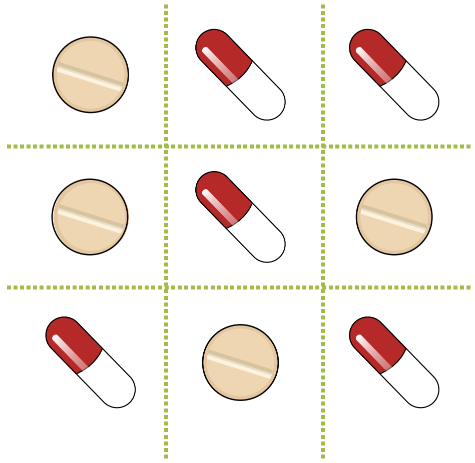

Mazes

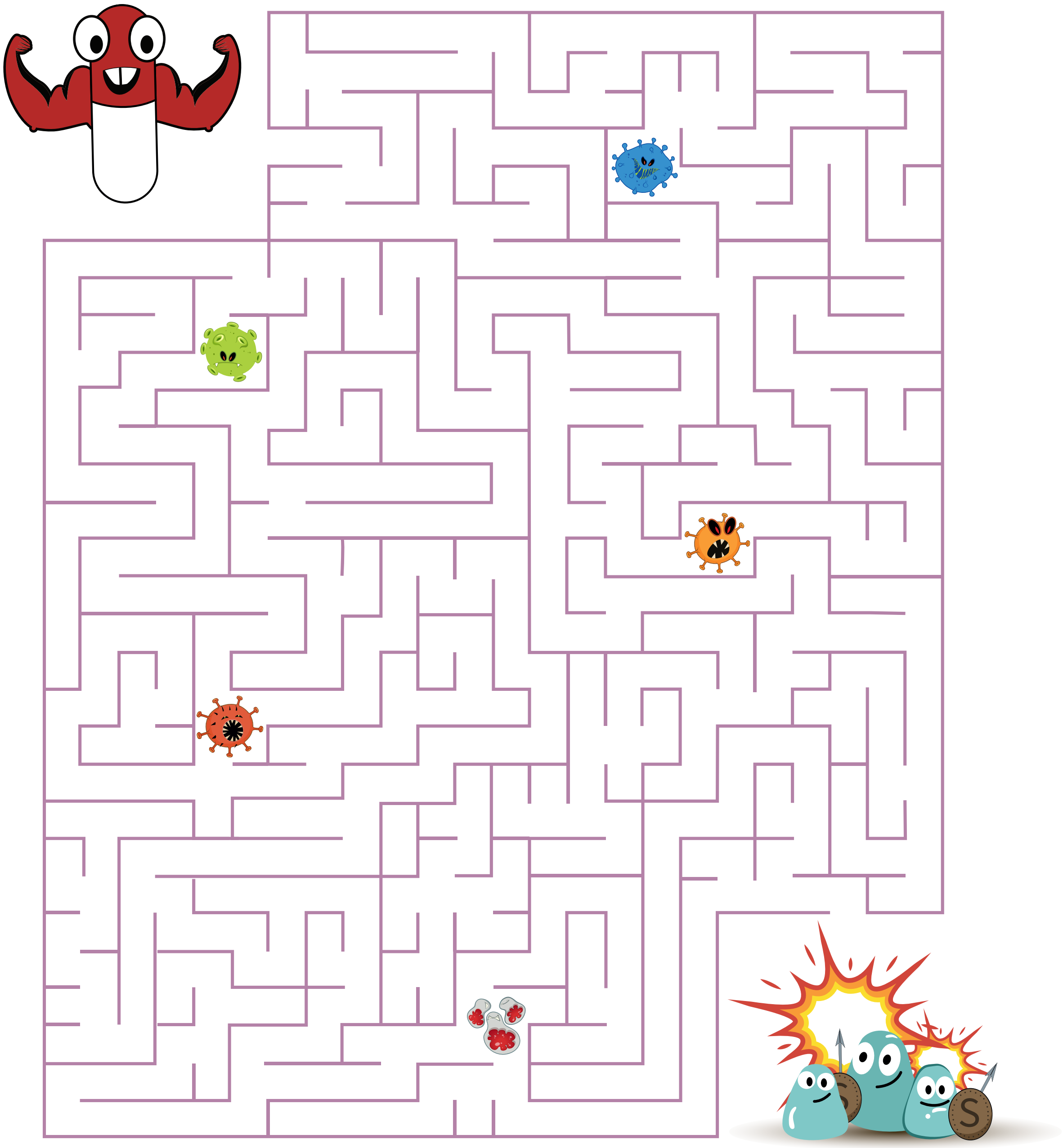

Notes

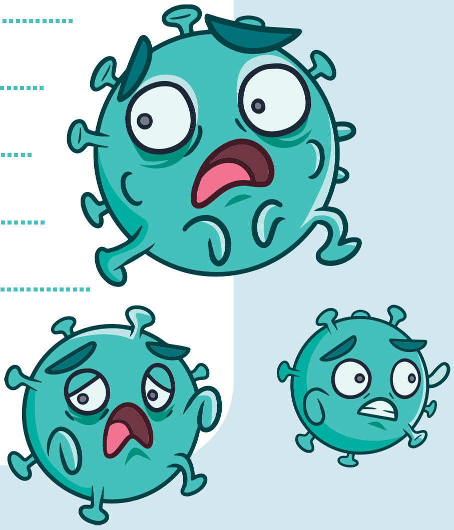

Notes

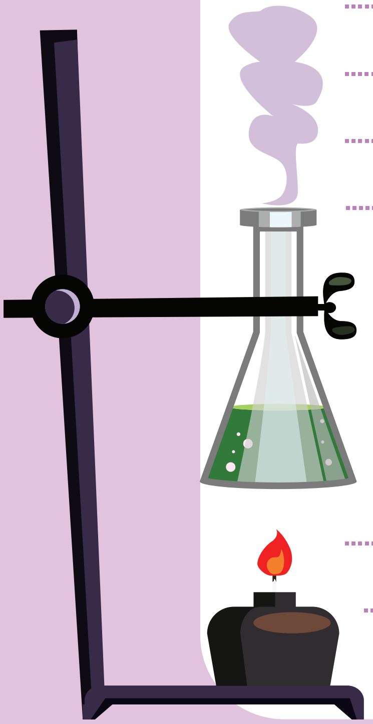

Notes

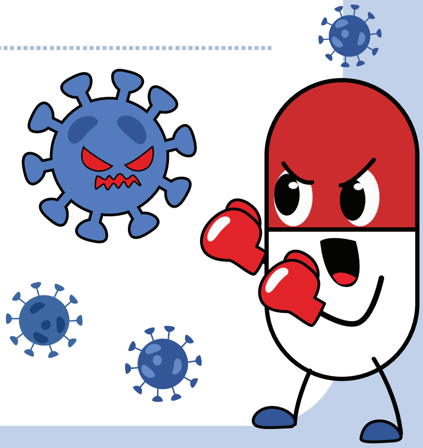

Notes

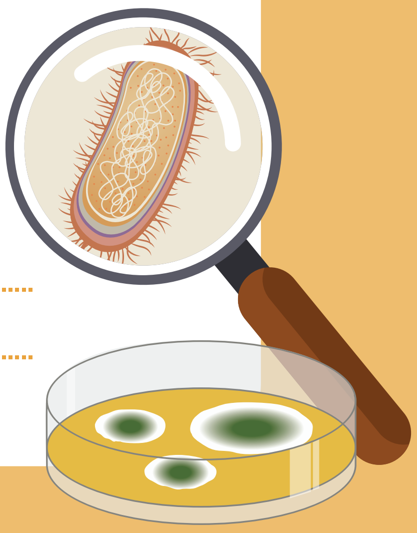

PUBLISHED BY

গণপ্রজাতন্ত্রী বাংলাদেশ সরকার

ঔষধ প্রশাসন অধিদপ্তর DGDA

FINANCIAL AND TECHNICAL SUPPORT:

Sweden
Sverige

World Health Organization
Bangladesh

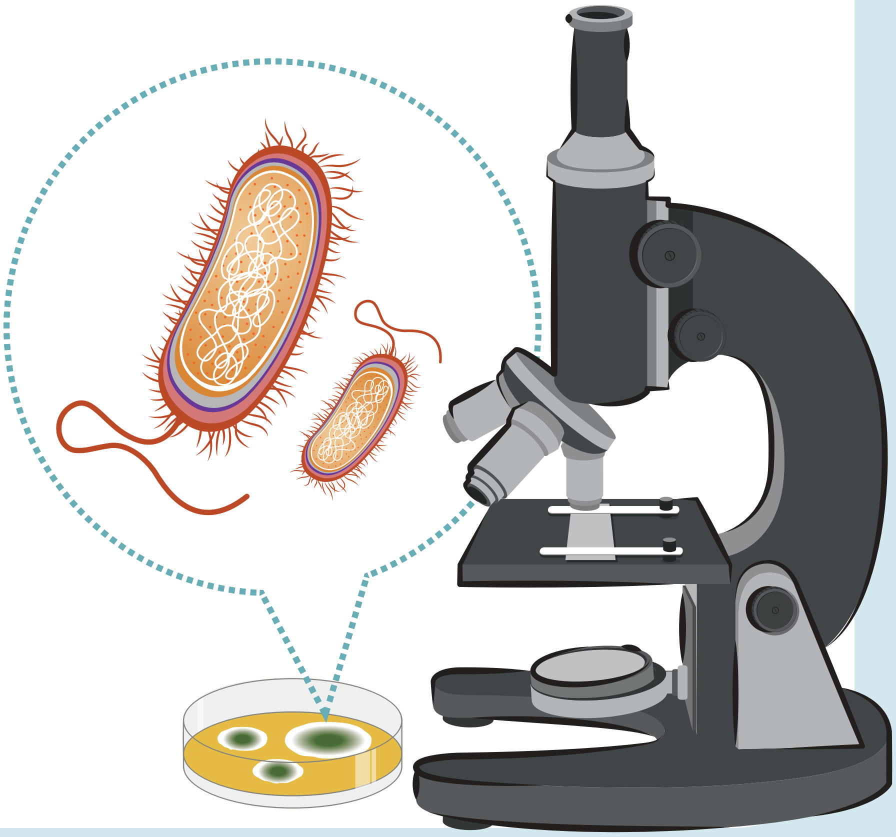

Supplement: Supplementary file 1 [file antibiotics-14-00979-s001.zip › File S3.pdf]
